# Wide-Temperature Electrolytes for Aqueous Alkali Metal-Ion Batteries: Challenges, Progress, and Prospects

**DOI:** 10.1007/s40820-025-01865-3

**Published:** 2025-08-11

**Authors:** Zichen Lin, Yongzhou Cai, Shilin Zhang, Jianguo Sun, Yu Liu, Yang Zheng, Kaifu Huo

**Affiliations:** 1 School of Metallurgy and Energy, State Key Laboratory of Advanced Refractories, Wuhan University of Science and Technology, Wuhan, 430081 People’s Republic of China; 2 School of Chemical Engineering, The University of Adelaide, Adelaide, 5000 Australia; 3 Department of Materials Science and Engineering, National University of Singapore, Singapore, 117574 Singapore; 4 Wuhan National Laboratory for Optoelectronics (WNLO), School of Optical and Electronic Information, Huazhong University of Science and Technology, Wuhan, 430074 People’s Republic of China

**Keywords:** Aqueous alkali metal-ion batteries, Wide-temperature electrolyte, Electrolyte regulation, Hydrogen bond networks

## Abstract

Aqueous alkali metal-ion batteries (AAMIBs) have been recognized as emerging electrochemical energy storage technologies for grid-scale applications owning to their intrinsic safety, cost-effectiveness, and environmental sustainability. However, the practical application of AAMIBs is still severely constrained by the tendency of aqueous electrolytes to freeze at low temperatures and decompose at high temperatures, limiting their operational temperature range. Considering the urgent need for energy systems with higher adaptability and resilience at various application scenarios, designing novel electrolytes via structure modulation has increasingly emerged as a feasible and economical strategy for the performance optimization of wide-temperature AAMIBs. In this review, the latest advancement of wide-temperature electrolytes for AAMIBs is systematically and comprehensively summarized. Specifically, the key challenges, failure mechanisms, correlations between hydrogen bond behaviors and physicochemical properties, and thermodynamic and kinetic interpretations in aqueous electrolytes are discussed firstly. Additionally, we offer forward-looking insights and innovative design principles for developing aqueous electrolytes capable of operating across a broad temperature range. This review is expected to provide some guidance and reference for the rational design and regulation of wide-temperature electrolytes for AAMIBs and promote their future development.

## Highlights

- The key challenges and fundamental principles of wide-temperature aqueous electrolytes for alkali metal ion batteries were analyzed.
- The design strategies for aqueous electrolytes with broad operating temperature ranges were summarized. The future research directions for high-performance wide-temperature aqueous alkali metal ion batteries were proposed.

## Graphical abstract

**Figure:**
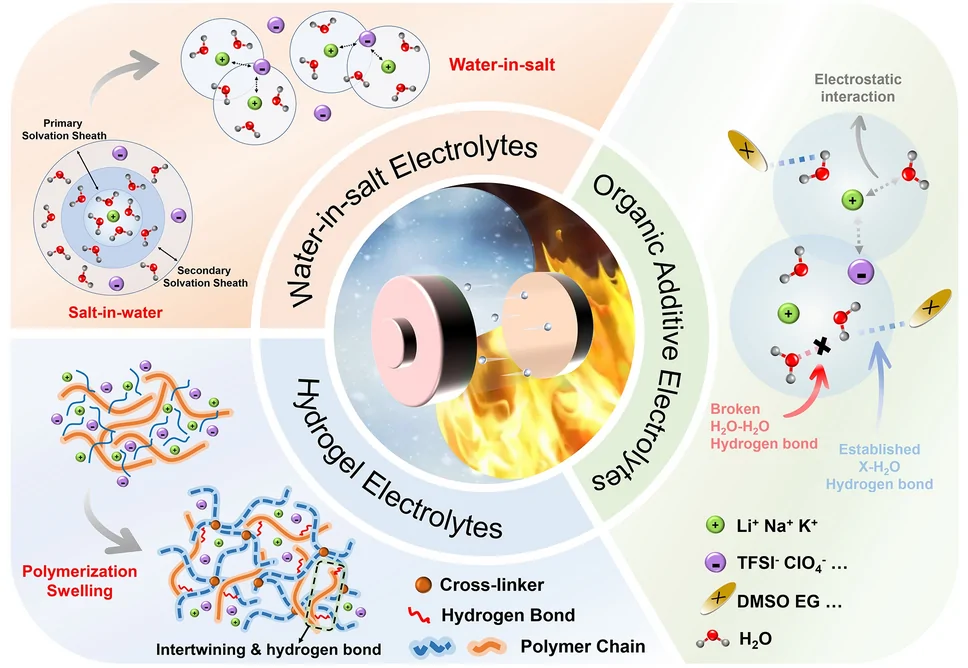

## Introduction

**Fig. 1:**
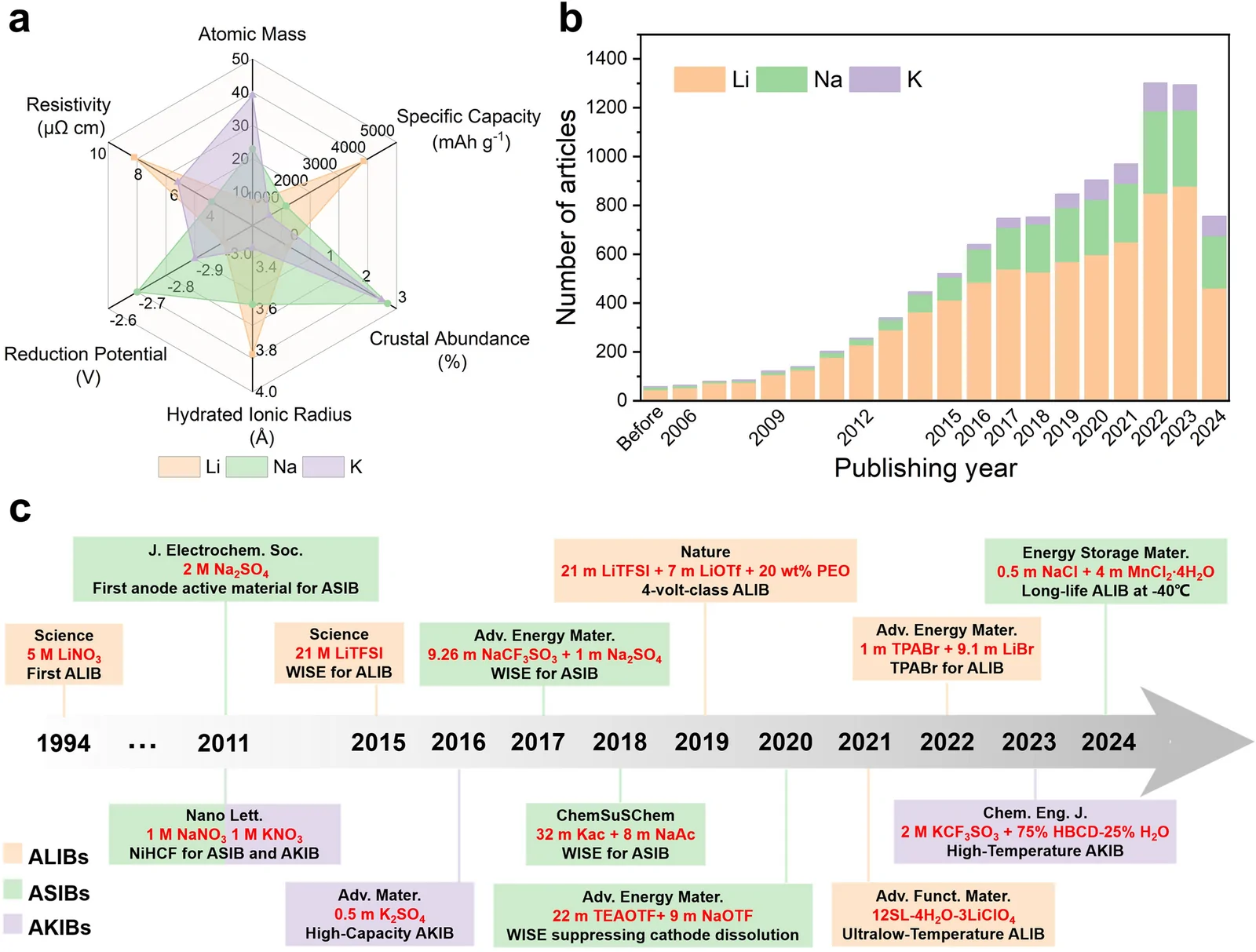
**a** Comparison of the physical properties of the alkali metal elements Li, Na, K. **b** Number of publications on “aqueous lithium/sodium/potassium ion batteries” (ALIBs, ASIBs, AKIBs), up to September 2024. Database: Web of Science. **c** Typical articles of aqueous alkali metal ion batteries in recent years

With the rapid development of human society, traditional fossil energy sources are gradually unable to meet the actual demand, so there is an urgent need to develop renewable energy sources and effective energy conversion systems [1]. Among various candidate technologies, electrochemical energy storage devices represented by rechargeable lithium-ion batteries have been widely used ranging from portable electronics to electric vehicles and greatly changed our lives due to their high energy density, long cycle life, and easy maintenance [2–4]. Over the past decade, other alkali metal-ion batteries (AMIBs), such as sodium and potassium ion batteries also have aroused widespread research interest because of similar mechanisms and rich resources [5–7]. Notably, sodium and potassium are far more abundant in the Earth’s crust (about 2.75% and 2.58%, respectively) compared to lithium’s 0.002%, making sodium and potassium-ion batteries more cost-effective and suitable for large-scale applications (Fig. 1a) [8]. Nevertheless, the flammability, toxicity, potential environmental pollution, and high cost of organic electrolytes used in commercialized AMIBs pose serious safety hazards and hinder the further development of grid-scale applications [9, 10]. Therefore, rationally exploiting novel electrolytes with intrinsic safety, affordability, and eco-friendliness is highly desired for numerous applications of AMIBs.

Aqueous alkali metal-ion batteries (AAMIBs) which use water as the electrolyte solvent have recently attracted tremendous attention due to their inherent merits of high safety, low cost, and environmental friendliness [11–15]. Compared with commercialized organic electrolytes, aqueous electrolytes can significantly enhance flame retardancy, endowing intrinsic safety, environmental benefits, and easy-to-manufacture of AAMIBs [16–19]. Furthermore, the radii of hydrated sodium and potassium ions are smaller than that of hydrated lithium ions (3.82, 3.58, and 3.31 Å for lithium, sodium, and potassium-hydrated ions, respectively), leading to higher conductivity in sodium and potassium-based aqueous electrolytes. This results in superior multiplicative properties for aqueous batteries [20, 21]. The trend in published articles related to these batteries, as shown in Fig. 1b, c, highlights the growing interest and research output in this field over recent years. Despite that, AAMIBs still face significant challenges for practical application. Particularly, temperature is an important factor affecting the performance, lifespan, and safety of aqueous batteries [22, 23]. As the demand for energy storage systems has grown and diversified in recent years, the ability of these devices to function under extreme weather conditions has become essential [24, 25]. For instance, electric vehicles require batteries that perform reliably in both cold and hot climates, and some medical devices must endure sterilization at wide-temperature range. Due to the high thermodynamic freezing point of water, the degradation of the electrochemical performance of aqueous batteries at low temperatures can be attributed to several factors: (I) increased electrolyte viscosity and slowed reaction kinetics, hindering ion transport; (II) impaired wettability of the electrode/electrolyte interface, leading to interface degradation and higher interfacial impedance; and (III) the electrolyte’s poor performance limiting the use of certain electrode materials across a wide temperature range [26–28]. In addition to low-temperature scenarios, these batteries also encounter high-temperature conditions such as over 40 °C, especially with the increasing severity of global warming [16]. While high temperatures can improve the reaction kinetics and ion transport in the electrolyte [22], they will also accelerate water evaporation and increase water activity. This exacerbates water decomposition side reactions, posing serious safety risks and reducing the safety and cycling stability of the batteries [24, 29]. High temperatures can also cause gas generation within the batteries, raising internal resistance and lowering output power. Thus, it is crucial to regulate electrolyte components to enhance water retention and reduce water activity to address these issues.

Previous reviews have mainly focused on the electrolyte design and performance optimization of aqueous batteries for low-temperature [27, 30–32], or ambient-temperature conditions [3, 14, 33, 34], while the advancement of designing high-temperature and wide-temperature electrolytes for AAMIBs is also very significant [35, 36]. Hence, in this review, we aim to present a systematic understanding of the challenges in electrolytes faced by the aqueous alkali metal ion batteries at wide-temperature range, while outlining the path forward. We provide a comprehensive overview of the development history and design strategies of wide-temperature-range aqueous electrolytes, highlighting their potential to overcome the limitations imposed by extreme temperature conditions. Finally, we offer our perspectives on future directions for advancing this technology.

## Basic Principles of Wide-Temperature Electrolytes for AAMIBs

### Low-Temperature-Resistant Aqueous Electrolytes

The electrolyte, which is central to ion transport, is vital in the electrochemical process of batteries [33, 37]. The change in the physical state of aqueous electrolytes at low temperatures closely relates to their composition, primarily because water is their solvent [32]. Since water freezes at 0 °C, lower temperatures cause the water to solidify, affecting the electrolyte’s performance. To understand the factors influencing low-temperature performance, it is essential to examine the microstructure of water molecules, as well as the thermodynamic and kinetic properties of the system.

#### Freezing Point of Water and Hydrogen Bonds

**Fig. 2:**
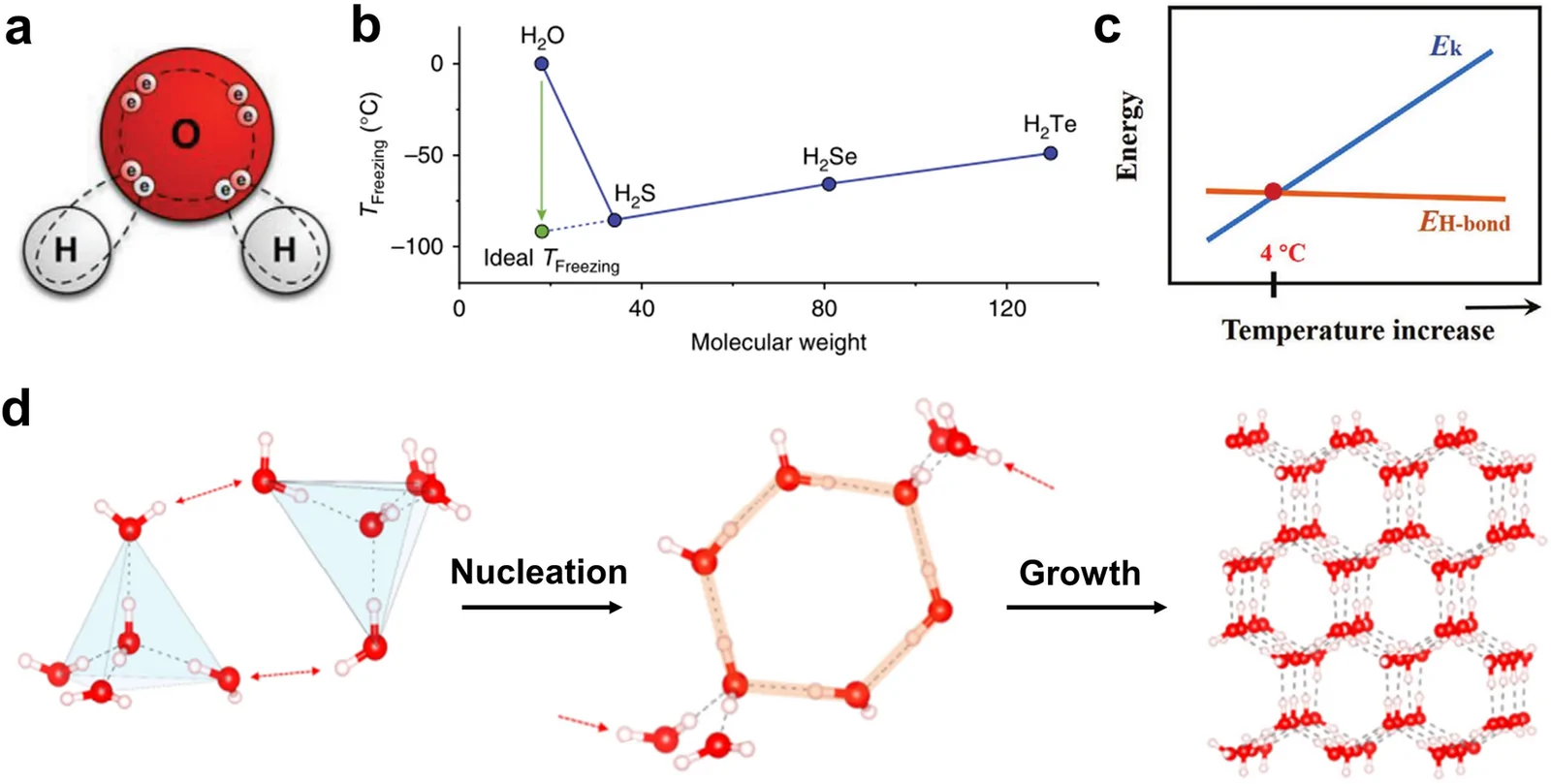
**a** Microstructure of water molecules. Adapted with permission form Ref. [32]. Copyright 2024, Wiley–VCH. **b** Freezing point of chalcogen hydrides. Adapted with permission form Ref. [43]. Copyright 2020, Springer Nature. **c** Temperature-dependent energy in pure water where E_k_ and E_H-bond_ refer to the kinetic energy and the energy of the H-bonds of water molecules. Adapted with permission from Ref. [30]. Copyright 2020, Wiley–VCH. **d** Ice nucleation and growth mechanism. Adapted with permission form Ref. [43]. Copyright 2020, Springer Nature

A water molecule is composed of one oxygen atom and two hydrogen atoms, which together create a stable structure with eight valence electrons (Fig. 2a). The oxygen ions and oxyanions in water have varying tendencies to acquire negative charges, leading to the formation of hydrogen bonds (H-bonds) between the O atoms and the H atoms of the neighboring H_2_O [38]. While not particularly strong, these H-bonds constantly form and break, creating a dynamic “hydrogen bond network” among the water molecules. It is well known that water exists in three physical states in nature, largely influenced by the presence and behavior of these H-bonds. Typically, up to four H-bonds can surround the O atom in a water molecule. Based on the strength and number of these bonds, water can be classified into three categories: strongly hydrogen-bonded water (SHW), weakly hydrogen-bonded water (WHW), and non-hydrogen-bonded water (NHW). The SHW, which has four H-bonds per molecule, corresponds to the state of “ice”; The NHW, with no H-bonds, corresponds to the state of “water vapor”; and WHW, the most common form, represents liquid water with an intermediate number of H-bonds [39]. Water molecules are in constant motion at the molecular level, with H-bonds forming and breaking in a dynamic equilibrium. Due to the abundance of H-bonds, this phenomenon significantly affects water’s macroscopic properties, including its anomalously high freezing point compared to other chalcogen hydrides (Fig. 2b) [40]. As the temperature drops below 4 °C, the average kinetic energy of water molecules decreases, which increases the probability of H-bond formation over bond breakage. This leads to greater bonding among water molecules in the system, resulting in a restriction of their motion (Fig. 2c). Eventually, when the temperature drops below freezing, locally short-range ordered liquid water is completely transformed into long-range ordered solid-phase ice [41]. Ice nucleation, as the initial stage of freezing, relies on water molecules with tetrahedral coordination structures growing into stacked hexagonal sequences [42] (Fig. 2d). The phase change of the solvent water in the electrolyte is a major factor contributing to the poor electrochemical performance of batteries in low-temperature environments. The microstructure and dynamics of the H-bonds between water molecules crucially influence the macroscopic properties, such as the freezing point of the aqueous solution [32, 43]. Therefore, it is important to reduce the solution’s freezing point by disrupting the H-bonds in water at low temperatures, thereby decreasing the amount of SHW.

**Fig. 3:**
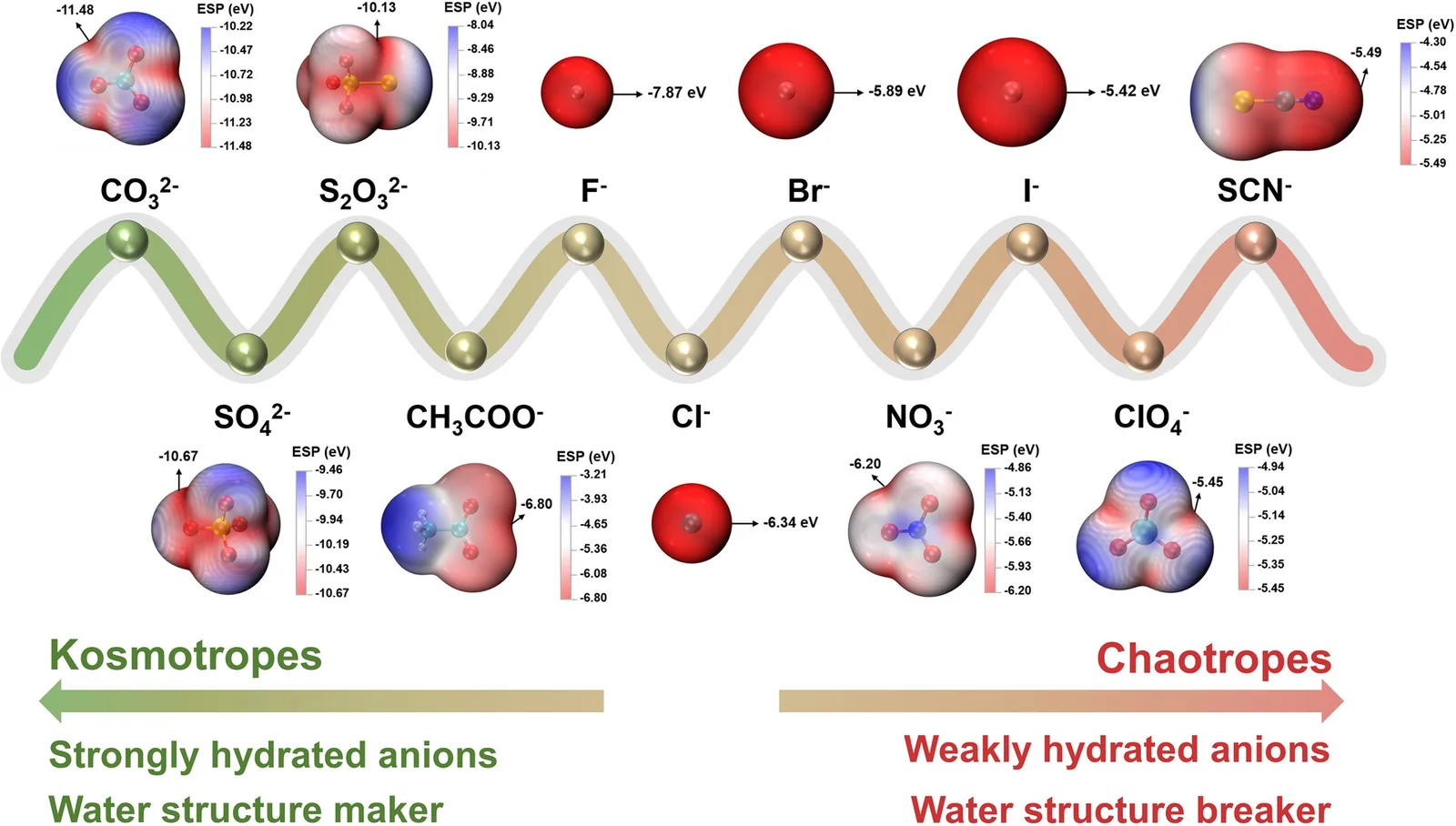
Hofmeister series and ESP of various anions from DFT calculations and MD simulations

The Hofmeister ion effect, first discovered by the German biochemist Hofmeister in the late nineteenth century, describes how different ions in solution can variably influence the dissolution and aggregation of solutes. In solution, ions can interact with other solutes and solvent molecules through electrostatic interactions, solvent molecular coordination, etc. However, not all ions affect solutes similarly, which is central to understanding the Hofmeister ion effect [44–46]. By leveraging this effect, specific ions can be selected to alter the coordination structure around water molecules, forming hydrated ions (also known as solvated ions) [47]. This process reduces the number of free water molecules in the solution and disrupts the ordered hydrogen bond network, particularly at low temperatures [48]. The degree of ion hydration is closely related to the ion’s surface charge density. Ions with low charge densities and large radii tend to have weaker hydration, leading to the disorder of the surrounding water molecules, and are known as “structure destroyers”. Conversely, ions with high charge densities are strongly hydrated, leading to a more orderly arrangement of water molecules and reduced thermal motion, and are termed “structure makers” [32, 49]. Anions generally exhibit more pronounced Hofmeister ionic effects than cations. The ordering of typical anions and their associated properties is illustrated in Fig. 3. While the origins of the Hofmeister series and the mechanisms underlying ion-specific effects remain complex and not yet fully understood, it has been suggested that an ion’s electronegativity influences its interactions with water molecules, thereby determining its placement within the Hofmeister series. To provide a clearer visualization of this relationship, the electronegativity of specific anions was assessed through density functional theory (DFT) calculations and molecular dynamics (MD) simulations. Ion hydration refers to the interactions between ions and surrounding water molecules, which significantly impact ion mobility, electrical conductivity, and other physicochemical properties. The degree of ion hydration is linked to electronegativity, as ions with higher electronegativity tend to attract more water molecules. Based on their hydration levels, anions can be classified into strongly hydrated kosmotropes and weakly hydrated chaotropes. Strongly hydrated ions promote H-bonds among water molecules, thereby reducing solution mobility and enhancing the structural organization of water. Conversely, weakly hydrated ions disrupt H-bonds, increasing solution mobility. These ions, often termed “structure-breakers”, weaken intermolecular H-bonds and destabilize the cohesive structure of water. In the Hofmeister series, kosmotropic ions are positioned to the left of chloride (Cl⁻), reinforcing water structure through strong H-bonds. In contrast, chaotropic ions are located to the right of Cl⁻ and disrupt the hydrogen bond network [50–52]. Consequently, selecting weakly hydrating ions can effectively modulate the hydrogen bond network in water, a critical consideration for applications requiring precise control over water’s physical properties.

#### Thermodynamic and Kinetic Interpretations

**Fig. 4:**
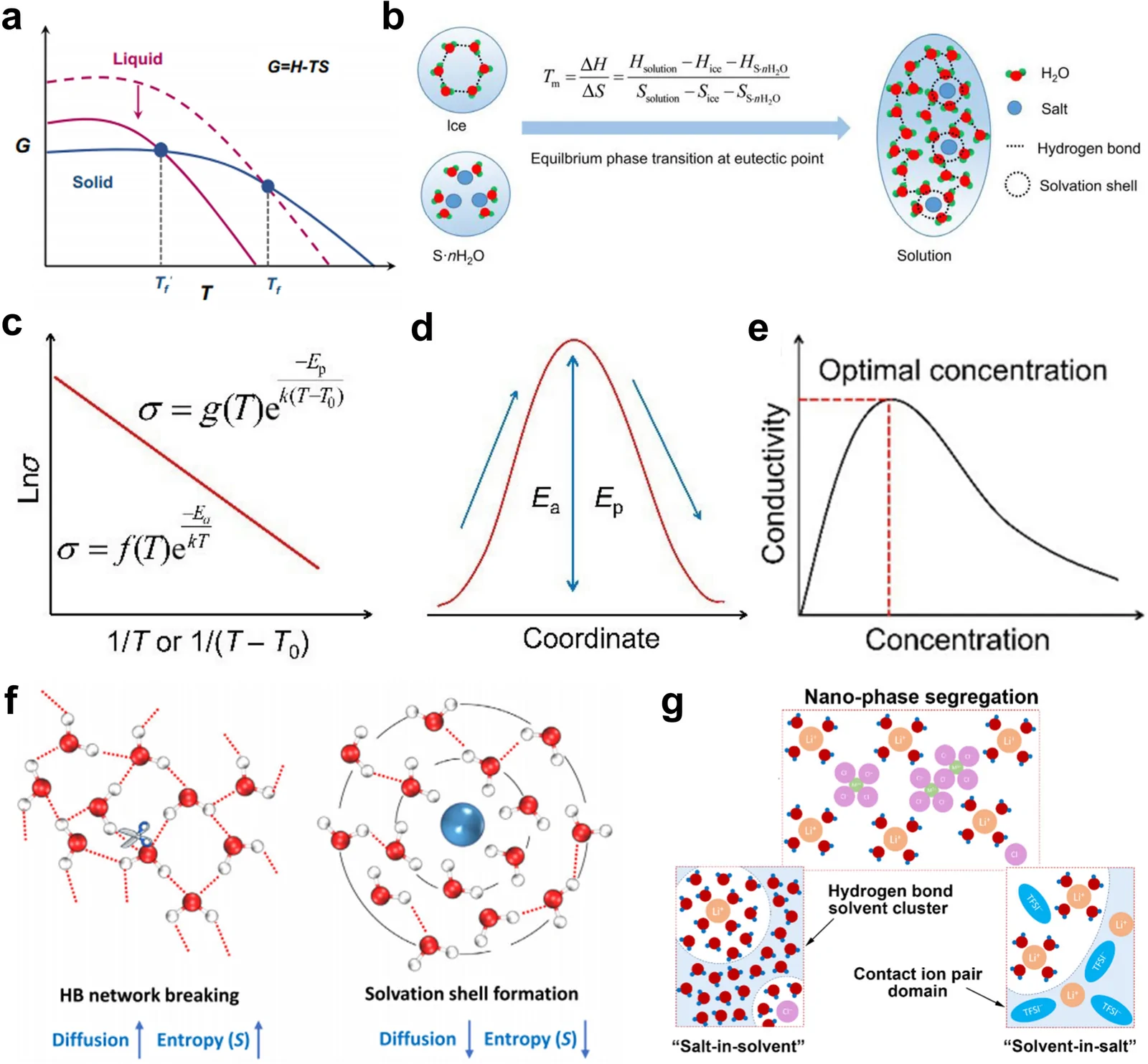
**a** Relationship between Gibbs free energy, the entropy of liquid, and the freezing point, in which *S* represents the entropy of systems. Adapted with permission from Ref. [53]. Copyright 2023, Springer Nature. **b** Schematic illustration of ice and hydrated salt S·nH_2_O transitioning to the solution at the eutectic point. The formula of phase transition temperature *T*_*m*_ is shown. **c** Schematic illustration of the temperature-dependent ionic conductivity. The insets are the typical Arrhenius and VTF equations. **d** Schematic illustration of the ions overcoming activation energy during the diffusion process. **e** Schematic illustration of the concentration dependence ionic conductivity. Adapted with permission from Ref. [54]. Copyright 2022, SciOpen. **f** Original water molecules form a tetrahedral network structure through H-bonds. When the network composed of tetrahedrally structured water is disturbed by the introduction of ions, the water diffusion dynamics and *S* increase simultaneously (left). In addition, a solvation shell forms around the ions. As a result of the restriction imposed by the ionic electric field, the diffusion dynamics of water molecules are slowed with a reduction in *S* (right). Adapted with permission from Ref. [53]. Copyright 2023, Springer Nature. **g** Illustration of solution structure in different electrolytes. Adapted with permission from Ref. [55]. Copyright 2023, Springer Nature

The liquid phase temperature of an electrolyte is thermodynamically determined by the Gibbs free energy difference between the solid and the liquid phases (Fig. 4a). This relationship is described by the following thermodynamic equation:

1 G=H-TS

where *G* (kJ mol^−1^) is the Gibbs free energy, *H* (kJ mol^−1^) is the enthalpy, and *T* (K) and *S* (J mol^−1^ K^−1^) are the temperature and entropy of the actual system [53]. At the solid–liquid transition temperature, the system reaches equilibrium, and the Gibbs free energy difference equals 0 [54]. The temperature at which this phase transition occurs is influenced by the ratio of enthalpy to entropy. The solution solid–liquid phase transition temperature depends on the enthalpy-to-entropy ratio during the phase transition process. Based on this, to lower the freezing point of the electrolyte, it is essential to adjust the enthalpy and entropy changes. Specifically, reducing the *H* and increasing the *S* will lower the solid–liquid transition temperature as denoted in Eq. (2).

2 $$T_{m} = \frac{\Delta H}{{\Delta S}} = \frac{{H_{{{\mathrm{solution}}}} - H_{{{\mathrm{ice}}}} - H_{{{\mathrm{S}} \cdot n{\mathrm{H}}_{2} {\mathrm{O}}}} }}{{S_{{{\mathrm{solution}}}} - S_{{{\mathrm{ice}}}} - S_{{{\mathrm{S}} \cdot {\mathrm{H}}_{2} {\mathrm{O}}}} }}$$

where *T*_*m*_ is the phase transition temperature; Δ*H* is enthalpy change; Δ*S* is entropy change; *H*_solution_, *H*_ice_, and $H_{{{\mathrm{S}} \cdot n{\mathrm{H}}_{2} {\mathrm{O}}}}$ are the enthalpies of the solution, ice, and hydrated salt S∙*n*H_2_O, respectively; *S*_solution_, *S*_ice_, and $S_{{{\mathrm{S}} \cdot n{\mathrm{H}}_{{2}} {\mathrm{O}}}}$ are the entropies of solution, ice, and S·*n*H_2_O, respectively [54]. Figure 4b shows the co-transformation of ice and the hydrated solute S∙nH_2_O into an aqueous solution at the eutectic point. During the phase transition, ice with strong H-bond interactions and S∙nH_2_O with strong electrostatic interactions become an aqueous solution with anionic and water interactions, contributing to the Δ*H*. Highly ordered ice and S∙*n*H_2_O form a disordered aqueous solution, contributing to the Δ*S*. According to Eq. (2), a lower Δ*H* and a higher Δ*S* lead to a lower *T*_*m*_. Currently used strategies to achieve these changes include adding inorganic salts, organic co-solvents, and hydrogels. These additives interact strongly with water, thereby decreasing the *H*. Additionally, due to the destruction of H-bonds, it also increases the *S*, which in turn reduces the solid–liquid phase transition temperature to improve the low-temperature performance [32, 54].

The viscosity, ionic conductivity, and solvation structure of the aqueous electrolyte significantly impact the low-temperature performance of aqueous batteries. The viscosity of the electrolyte increases due to the spontaneous increase in salt concentration. From a kinetic perspective, the viscosity, ionic conductivity, and solvation structure of the aqueous electrolyte are also key factors that affect the low-temperature performance of aqueous batteries. As temperature decreases, the electrolyte viscosity of aqueous solutions increases, due to the spontaneous increase in salt concentration when free water crystallizes into ice. Adding a suitable low melting point non-polar solvent to the aqueous electrolyte makes it possible to reduce its viscosity at low temperatures. However, this adjustment also decreases the total dielectric constant and reduces the number of available charge carriers, consequently lowering the ionic conductivity [30, 55–57]. The temperature dependence ionic conductivity in liquids generally follows the Arrhenius equation or Vogel–Tammann–Fulcher (VTF) equation in Eqs. (3) and (4), respectively.

3 $$\sigma = f\left( T \right)e^{{ - \frac{{E_{{\mathrm{a}}} }}{kT}}}$$

4 $$\sigma = g\left( T \right)e^{{ - \frac{{E_{{\mathrm{p}}} }}{{k\left( {T - T_{0} } \right)}}}}$$

where *σ* is the ionic conductivity; *T* is the temperature; *f*(*T*) is the pre-exponential factor, which is a constant but sometimes contains a factor 1/*T*; *E*_a_ is the activation energy; *k* is the Boltzmann constant; *g*(*T*) is the pre-exponential factor, which is a constant but sometimes contains a factor $1/\sqrt{T}$; *T*_0_ is the ideal glass transition temperature; and the *E*_p_ is the pseudo activation energy [54].

The Arrhenius and VTF equations are useful for predicting the ionic conductivity at different temperatures as shown in Fig. 4c, d. The activation energy is obtained by fitting the temperature-dependent ionic conductivity, which allows comparison of ion diffusion kinetics in different systems. Lower activation energy indicates faster ion diffusion kinetics [54]. As shown in Fig. 4e, the ionic conductivity of the electrolyte typically rises to a peak and then declines as salt concentration increases. In addition, when “structure-breaking” salts are introduced into water, the hydrogen bond network between water molecules is usually disrupted by dipole–dipole interactions, leading to faster diffusion kinetics and higher entropy (*S*) (Fig. 4f, left). However, some water molecules form a solvated shell around the ions, which restricts the movement of the ions to a certain extent, thus slowing down the diffusion kinetics (Fig. 4f, right). At the same time, high salt concentrations play a role in disrupting the intermolecular network in free solvent clusters (Fig. 4g). The solvent activity is reduced thereby inhibiting the contraction and crystallization of solvent clusters at low temperatures [43, 58]. However, the amount of hydrated water will also increase with the increase in the solute concentration. If the concentration becomes too high, the interaction between water molecules and ions diminishes due to increased coupling between anions and cations. This interaction can lead to solute precipitation as the temperature drops [31, 59, 60]. Thus, a carefully managed increase in salt concentration can enhance the electrochemical performance of aqueous electrolytes at low temperatures.

### High-Temperature-Resistant Aqueous Electrolyte

The greatest advantage of aqueous electrolytes over current organic-based flammable ester electrolytes is their high safety [61]. However, compared to broadening the low-temperature operating temperature, the expansion of the high-temperature operating temperature of aqueous batteries is more difficult due to the exponential increase in saturated water vapor pressure at elevated temperatures [62]. Therefore, operating at high temperatures presents challenges such as accelerated ion diffusion, electrolyte/electrode voids, and water evaporation [63, 64]. The problem faced by aqueous batteries at high temperatures is to ameliorate the accelerated hydrogen evolution from the water and the undesirable byproducts, which are due to the increased electrochemical activity of water [65]. This leads to a narrowing of the electrochemical window. Strategies to suppress water activity and widen the electrochemical window are essential to enhance the high-temperature tolerance of aqueous batteries. This can involve developing electrolytes that are water-retentive and thermally stable, as well as modifying the electrodes to reduce side reactions, particularly at the anode, caused by increased water activity [54, 66, 67].

The key to developing high-temperature aqueous batteries lies in strengthening the covalent bond within water molecules and inhibiting water activity. This involves redesigning the hydrogen bond network, eliminating solvated water, and reducing water content [68–70]. Theoretically, hydrogel electrolytes can stabilize water by preventing the movement of free water at high temperatures, with their abundant polar groups providing a large number of anchoring sites for the free water [71, 72]. Disrupting the hydrogen bond network between water molecules helps achieve stabilization. In practice, however, hydrogel electrolytes tend to evaporate rapidly at high temperatures, leading to a loss of flexibility and ionic conductivity. Additionally, water loss typically causes the hydrogel to shrink, creating spatial separation between the hydrogel electrolyte and the electrodes, which increases interfacial resistance and degrades cycling performance and service life [73, 74]. Consequently, most AAMIBs reported to date cannot operate at high temperatures, with electrolyte evaporation remaining a significant obstacle to their development.

Studies have shown that replacing H₂O molecules in cationic sol–gel sheaths with strongly soluble co-solvents can effectively suppress water-related side reactions [75]. However, this approach often leads to high activation energy for desolvation and the formation of non-ionic conductive solid electrolyte interfacial (SEI) phase substances due to co-solvent decomposition. Strong solvent molecules tend to displace water molecules from the solvation sheath due to spatial site-barrier effects, resulting in an increase in free water molecules in the electrolyte. As a result, this modification strategy generally fails to enhance the chemical stability and temperature resistance of the electrolyte [76]. Alternatively, a weak-solvent modulation strategy, used in lithium-metal batteries, involves using a solvent that acts as a “diluent” that does not participate significantly in the formation of the inner solvent sheath [77] Although this approach has not been extensively studied in aqueous batteries, it is proposed that strong interactions between a weakly solvated electrolyte and H_2_O can promote the formation of a locally concentrated electrolyte. This concentration rebuilds the hydrogen bond network, thus preventing water decomposition and extending the electrolyte’s operating temperature range [78, 79].

### Wide-Temperature-Resistant Aqueous Electrolyte

The fundamental mechanisms for enhancing both low- and high-temperature electrolyte performance have been outlined above. In contrast, wide-temperature aqueous electrolytes require optimized design to maintain stability over an extended temperature range. While modifications targeting either low- or high-temperature stability focus on solvation structure optimization, wide-temperature electrolyte development necessitates fine-tuning solvent–solute interactions to modify physicochemical properties. For example, strengthening interactions between specific ions and water molecules inhibits H-bond formation, thereby lowering the freezing point. This strategy applies to both low-temperature and wide-temperature electrolyte modifications [80]. Additionally, interfacial stability is crucial. At low temperatures, electrolytes must prevent water freezing while maintaining ionic conductivity, whereas at high temperatures, water decomposition and side reactions must be suppressed. In wide-temperature electrolytes, ensuring stability at both temperature extremes is essential. The principal mechanisms influencing wide-temperature aqueous electrolytes include:

1. Temperature Adaptability of the Solvated Structure: Adjusting solvation structures enables electrolytes to function across varying temperatures. For instance, strong interactions between anisole (AN) and 2-methyltetrahydrofuran (MeTHF) can suppress parasitic reactions at high temperatures, while the combination of anisole (AN) and tetrahydrofuran (THF) prevents salt precipitation at low temperatures. Similarly, carefully selected additives and ionic interactions can enhance electrolyte stability over a wide temperature range [37].
2. Balancing Thermal Stability and Low-Temperature Performance: At high temperatures, increased water activity exacerbates decomposition reactions, impairing cycling performance and safety. Conversely, at low temperatures, electrolyte freezing reduces ionic conductivity and diminishes battery performance. Minimizing the activity of free water mitigates these issues. Introducing thermally stable salt additives or co-solvents enhances electrolyte performance across the temperature spectrum [35].
3. Formation and Stability of the Interfacial Film: A well-formed interfacial film stabilizes the electrolyte–electrode interface at extreme temperatures. Yue et al. [81] demonstrated that LiTFSI aqueous solutions exhibit strong interactions with CO_2_, leading to a CO_2_-rich electrolyte system. The reduction of Li_2_CO_3_ at the negative electrode forms a protective SEI film, broadening the electrolyte’s electrochemical window and improving stability across temperature extremes.
4. Synergistic Optimization of Electrode Materials: The successful implementation of wide-temperature electrolytes requires not only electrolyte modifications but also complementary electrode material optimizations. For example, designing electrode materials with high conductivity and robust interfacial stability further enhances battery performance across a broad temperature range [80].

## Low- and High-Temperature Electrolyte Design Strategy for AAMIBs

Among various aqueous energy storage systems, AAMIBs have emerged as a promising alternative to lithium-ion batteries. However, AAMIBs encounter several challenges that limit their practical application, including a restricted operating temperature range, a narrow electrochemical stabilization window (ESW) of the electrolyte, and the dissolution of cathode materials [82, 83]. To overcome these challenges, various electrolyte engineering strategies have been proposed, such as water-in-salt electrolytes (WISE) [43, 53], organic cosolvent hybrid electrolytes [84–86], and hydrogel electrolytes [87, 88]. Overall, the key challenges in the design and operation of electrolyte materials for these batteries can be categorized as follows:

1. Electrolyte Freezing at Low Temperatures: The high freezing point of water causes electrolyte solidification at low temperatures, significantly reducing ionic conductivity, increasing charge/discharge polarization, and impairing battery operation.
2. Side Reactions and Safety Issues at High Temperatures: Elevated temperatures increase water activity, intensifying decomposition reactions and leading to reduced battery cycling performance and potential safety risks such as expansion and thermal runaway.
3. Limited Electrochemical Stability Window: The low thermodynamic decomposition voltage of water (1.23 V) restricts the electrochemical stability window of aqueous electrolytes, limiting the battery’s output voltage and energy density.
4. Stability and Compatibility of the Electrode–Electrolyte Interface: The formation of a solid electrolyte interphase (SEI) significantly influences battery performance. At extreme temperatures, poor electrolyte wettability increases interfacial impedance, negatively affecting electrochemical performance.
5. Selection and Design Constraints of Electrode Materials: Water’s high polarity, strong coordination ability, and hydrogen evolution tendencies restrict the choice of electrode materials and exclude low-cost current collectors such as aluminum foil.

**Fig. 5:**
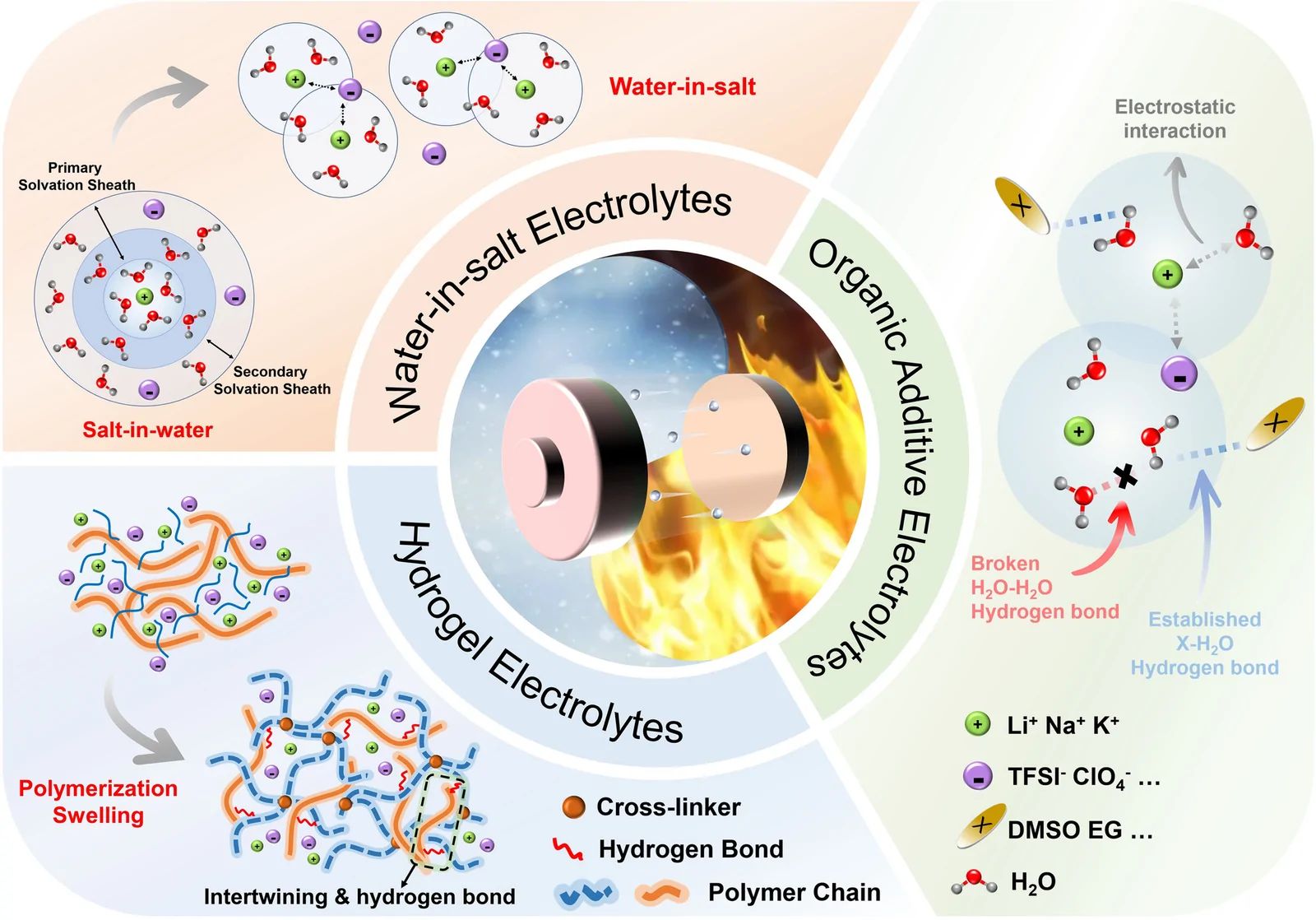
Design strategy for aqueous alkali metal ion battery electrolyte at wide temperatures

This section will focus on the development of designing wide-temperature range electrolytes for AAMIBs, summarize recent representative strategies to improve the performance across a wide temperature range (Fig. 5), and offer insights for future research directions.

### Low-Temperature Design Strategy

#### Salt Regulation Strategy

Aqueous batteries have shown a broad application prospect in the field of energy storage because of their high safety and environmental friendliness. However, the ionic conductivity of electrolytes decreases significantly at low temperatures, and the electrode reaction kinetics is slow, which seriously limits its practical application. In recent years, controlling solvation structure through electrolyte engineering has become an effective strategy to improve low-temperature performance. Among them, the salt regulation strategy can effectively reduce the freezing point of the electrolyte by optimizing the type and concentration of salt, improving ion mobility, and providing a new idea for improving low-temperature performance [89, 90]. Common used cations include polyvalent cations such as Zn^2+^ [91], Mg^2+^ [92, 93], and Ga^2+^ [39], while typical anions are halogens like Br^−^ [94], Cl^−^ [95], etc., as well as perchlorate ions (ClO_4_^−^ [96]).

**Fig. 6:**
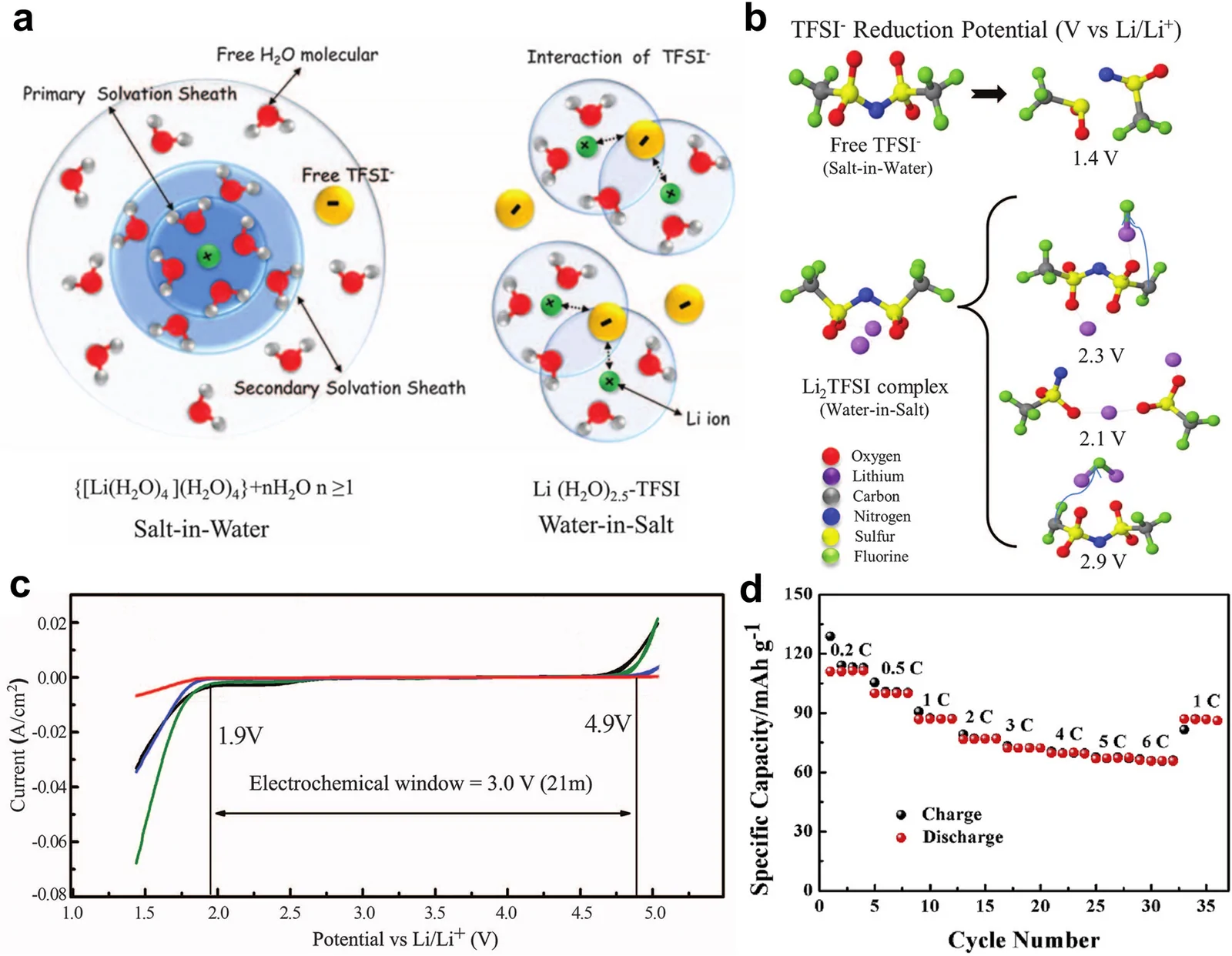
**a** Schematic evolution of Li^+^ primary solvated sheaths in dilute solution and “WISE”. Adapted with permission **b** Predicted reduction potentials from G4MP2 quantum chemistry calculations. **c** Wide electrochemical stability window for different concentrations of LiTFSI aqueous electrolytes. Adapted with permission from Ref. [97]. Copyright 2015, The American Association for the Advancement of Science. **d** Multiplier performance for LVP/LTP aqueous lithium-ion battery with 21 M LiTFSI as electrolyte at − 20 °C. Adapted with permission from Ref. [100]. Copyright 2018, Elsevier

To address the issue of the narrow electrochemical stability window (ESW) of water (1.23 V), Suo et al. [97] first introduced the “WISE” (water-in-salt electrolyte) concept in 2015. Molecular dynamics simulations reveal the relationship between solution structure and electrochemical properties. For dilute solutions, Li^+^ remains well hydrated in the primary solvation sheath. Since the anodic lithiation potential is lower than the reduction potential of water and sustained hydrogen evolution, it will organize the insertion of Li^+^ and the reduction of TFSI^−^. When in ultra-high-concentration LiTFSI solutions, two TFSI^−^ (Fig. 6a) are observed on average in each Li^+^ primary solvation sheath, and such a high probability of TFSI^−^ leads to an interfacial chemistry dominated by the reduction of TFSI^−^. The reduction potential of TFSI^−^ is also altered by its interaction with Li^+^. According to DFT calculations (Fig. 6b), aggregates such as Li_2_(TFSI)(H_2_O)_x_ become reductively unstable below 2.9 V, which is considerably higher than the reduction potential and hydrogen precipitation potential of TFSI^−^. Therefore, in high-concentration LiTFSI solutions, the reduction process generates sufficient LiF from TFSI^−^ to form anodic electrolyte interphase, which kinetically prevents the continuous reduction of water and TFSI^−^, thus improving the electrochemical stability of the electrolyte [98, 99]. They developed a 21 M (mol L^−1^) LiTFSI with ultra-high concentration for aqueous lithium-ion batteries, which altered the Li^+^ solvation structure and extended the electrochemical stability window to 3 V (Fig. 6c). Since then, extensive research has leveraged the “WISE” concept to extend the electrochemical stability window by reducing the free water content in the solution through a higher salt concentration. At the same time, this strategy also lowers the freezing point of the aqueous solution, enhancing low-temperature performance and broadening the operating temperature range. For instance, Wang et al. [100] used 21 M LiTFSI to create a rechargeable aqueous lithium-ion battery with all-NASCION cathode material. Thanks to the low freezing point, high electrolyte concentration, and high ionic conductivity of the all-NASCION electrode material, the battery demonstrated excellent electrochemical performance at − 20 °C with a discharge rate of 0.2 C, the full battery achieved a high reversible capacity of 111 mAh g^−1^ (91% room temperature capacity), which maintains 60% (66.7 mAh g^−1^) capacity when current density was increased to 6 C (Fig. 6d).

**Fig. 7:**
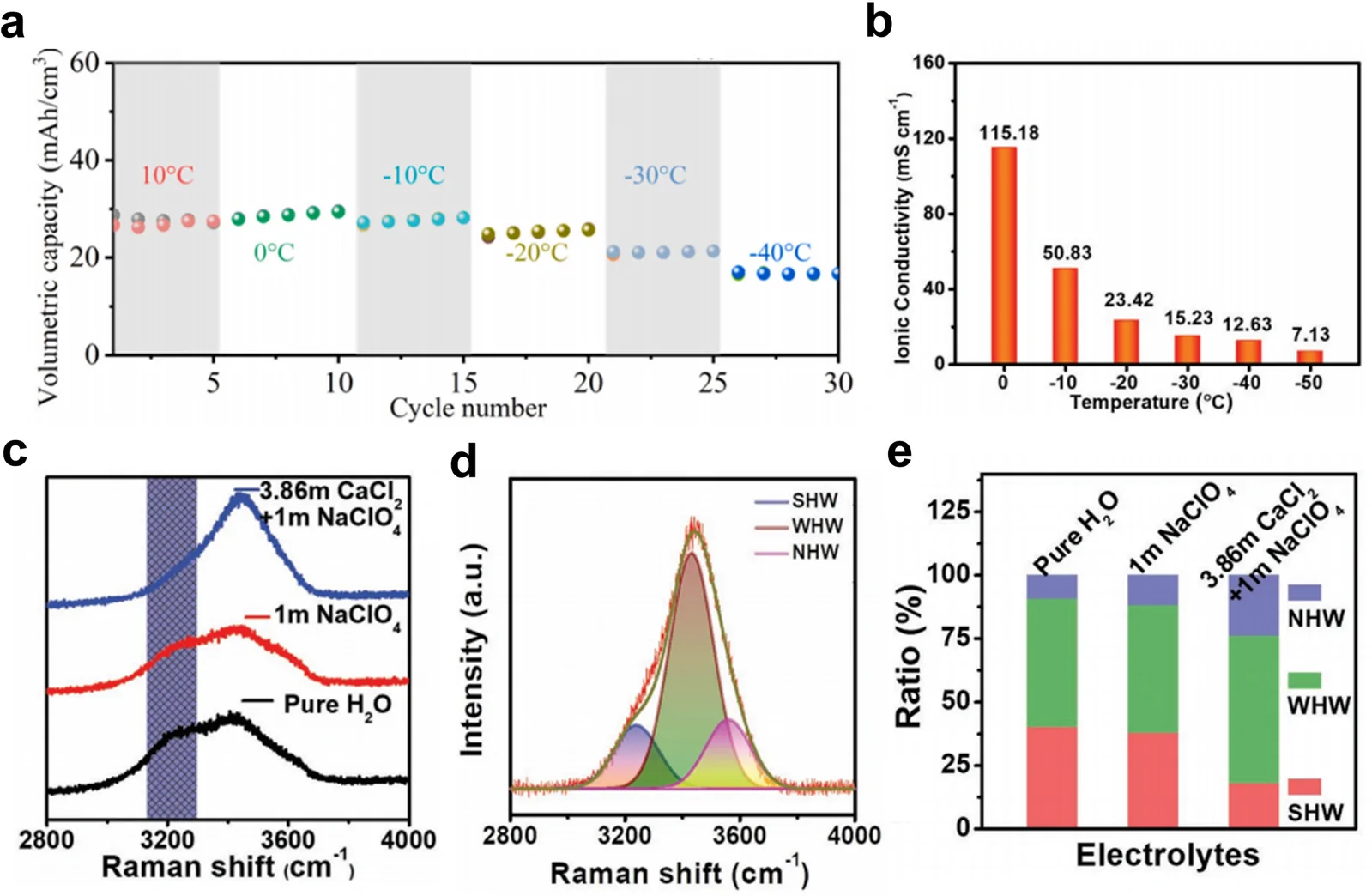
**a** Cyclability of the NVP//NaClO_4_//NVP aqueous sodium-ion batteries was measured at different temperatures ranging from 10 to − 40 °C at 0.3 mA cm^−2^. Adapted with permission from Ref. [103]. Copyright 2020, Elsevier. **b** Ionic conductivity of 3.86 m CaCl_2_ + 1 m NaClO_4_ electrolyte at different temperatures. **c** Raman spectra results of NaClO_4_-based electrolytes. **d** Fitted O–H stretching vibration in the Raman spectra for 3.86 m CaCl_2_ + 1 m NaClO_4_ electrolyte. **e** Component proportions of water with different H-bonds for various electrolytes: NaClO_4_-based electrolytes. Adapted with permission from Ref. [39]. Copyright 2022, Wiley–VCH

Compared with Li, Na offers the advantages of greater abundance and more stable electrochemical performance [101, 102]. As a promising renewable and sustainable energy storage technology, aqueous sodium-ion batteries have increasingly attracted scholarly attention [20, 103, 104]. In conjunction with the Hofmeister series, ClO_4_^−^ is regarded as a chaotrope (water structure breaker) that can disrupt water structures [52], which theoretically lowers the freezing point of the solution [105]. Wang et al. [106] employed a highly concentrated 17 m (mol kg^−1^) NaClO_4_ solution, enabling an aqueous sodium-ion battery system with a high capacity of 16 mAh cm^−3^ and a retention rate of 88% at − 40 °C (Fig. 7a). While a high concentration of electrolyte can be effective widen the electrochemical stabilization window and lower the freezing point, excessive salt concentration not only increases costs and the viscosity but also hinder ion transport and pose potential safety risks [107, 108]. To mitigate these challenges, researchers have explored dual-salt electrolytes to achieve improved low-temperature performance at a lower cost. Zhu et al. [39] enhanced the low-temperature performance of an electrolyte by adding the inexpensive inert additive CaCl_2_ solution. The optimized electrolyte achieved a freezing point even below − 100 °C and exhibited high ionic conductivity (7.13 mS cm^−1^) at − 50 °C (Fig. 7b). It can be seen from the Raman spectrum (Fig. 7c-e) reveals a significant decrease in the amount of SHW in the optimized electrolyte and an increase in the amount of WHW and NHW indicating that CaCl_2_ can disrupt the structure of the aqueous solution. While the study focused on the role of Ca^2^⁺, the introduction of Cl^−^ was not analyzed, and this aspect could be explored in future research.

**Fig. 8:**
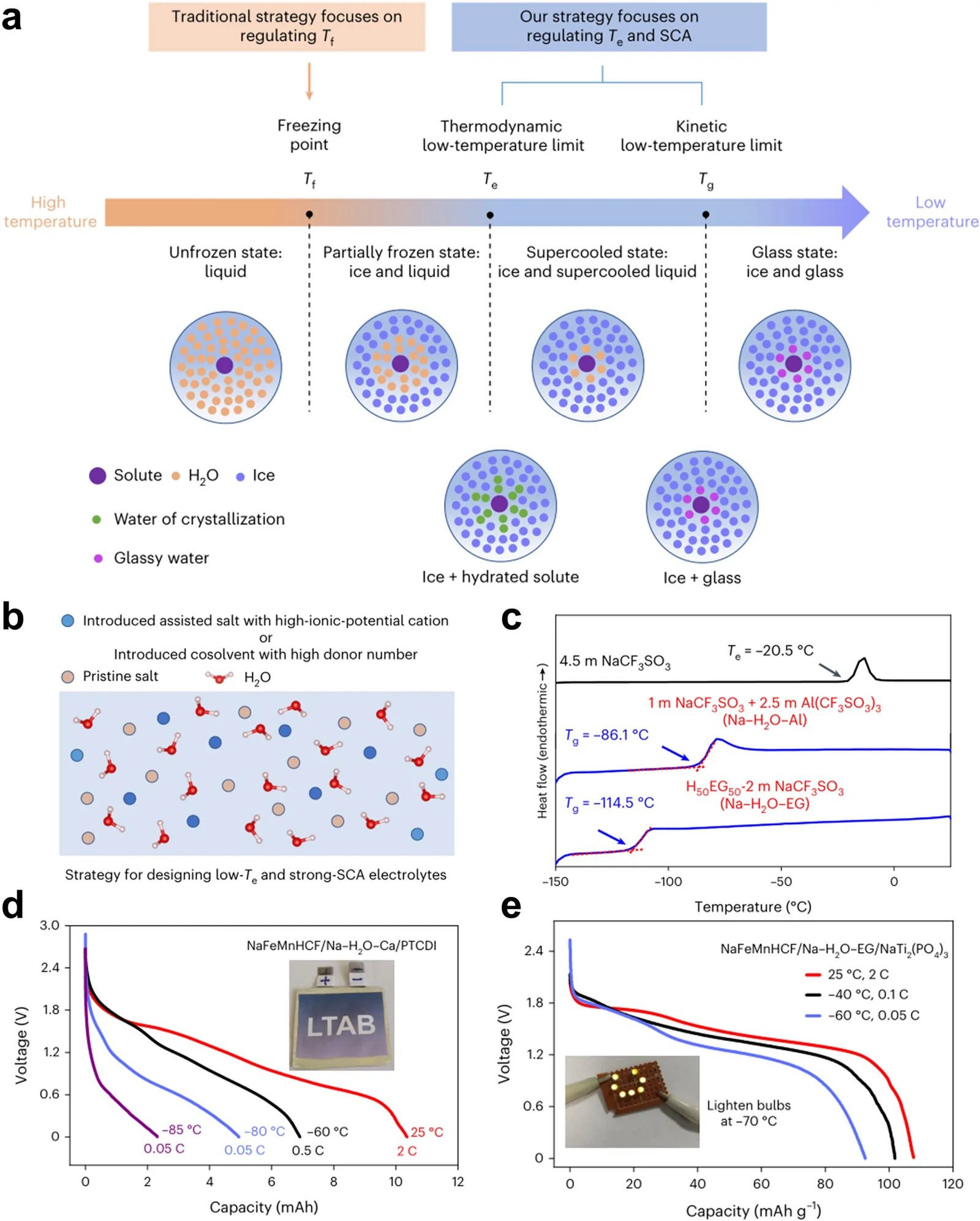
**a** Schematic evolution of a dilute solution in the H_2_O-solute system during the cooling process and the difference between traditional and our proposed strategies. **b** Schematic illustration of the proposed general strategy for designing low-*T*_*e*_ and strong-SCA electrolytes. **c** DSC heating curves of 4.5 m NaCF_3_SO_3_ electrolytes, 1 m NaCF_3_SO_3_ + 2.5 m Al(CF_3_SO_3_)_3_ (Na-H_2_O-Al) and H_50_EG_50_-2 m NaCF_3_SO_3_ (Na-H_2_O-EG) electrolytes. **d** Typical discharge curves of NaFeMnHCF/Na-H_2_O-Ca/PCDI pouch cell at temperatures ranging from − 85 to 25 °C. **e** Cycling stability of a NaFeMnHCF/Na-H_2_O-Ca/PCDI coin cell at − 80 °C and 0.1 C. Adapted with permission from Ref. [109]. Copyright 2024, Springer Nature

Three terms are generally involved in low-temperature aqueous systems: freezing point (*T*_f_), eutectic temperature (*T*_e_), and glass transition temperature (*T*_g_). Most researchers have focused on regulating the electrolyte *T*_f_ by various methods. However, Jiang et al. [109] suggested that *T*_f_ is not the most important temperature-limiting factor for low-temperature batteries. As shown in Fig. 8a, *T*_f_ is the temperature at which the electrolyte begins to freeze and is in a partially frozen state, and the water-containing electrolyte can enable the battery to operate at temperatures lower than *T*_f_. At temperatures below *T*_f_, the diluted electrolyte typically becomes a mixture of ice and concentrated electrolyte, and the connected liquid regions can still maintain a sufficiently high ionic conductivity. In contrast, *T*_e_ is the temperature at which the electrolyte is completely frozen. It is critical to design freeze-proof electrolytes by selecting low *T*_e_ aqueous solute systems for extremal low-temperature applications. The *T*_e_ value of the solution can be reduced by increasing the number of solutes, so they proposed to create multi-solute systems by introducing salts with high ionic potential cations or co-solvents with high donor numbers (Fig. 8b). Therefore, they designed a variety of solute system electrolytes by introducing auxiliary salts with high ionic-potential cations or co-solvents with high donor numbers with ultra-low *T*_e_ (− 53.5 to − 72.6 °C) and *T*_g_ (− 86.1 to − 117.1 °C) (Fig. 8c). As shown in Fig. 8d, e, taking the Na-based system as an example, the designed electrolyte of 1 m NaClO_4_ + 4 m Ca(ClO_4_)_2_ (Na-H_2_O-Ca) can still operate at − 85 °C in a full cell, and the full cell with H_50_EG_50_-2 m NaCF_3_SO_3_ (Na-H_2_O-EG) as the electrolyte achieved an energy density of 63 Wh kg^−1^ at − 60 °C and 0.05 °C.

**Fig. 9:**
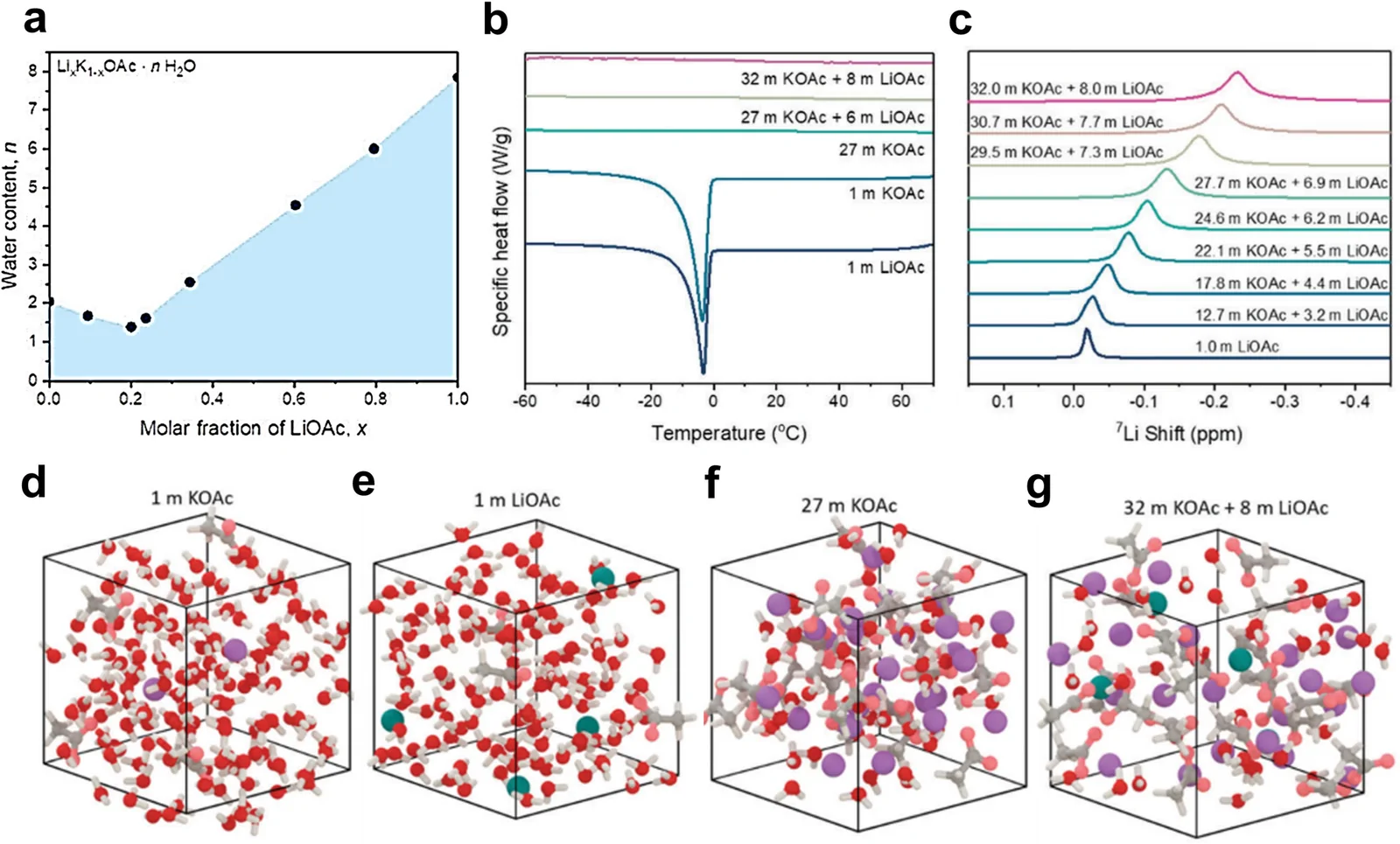
**a** Measured liquidus line of the Li_x_K_1−x_OAc salt-water mixtures. **b** Vertically offset differential scanning calorimetry data of solutions collected at a rate of 1 °C min^−1^. **c** ^7^Li NMR spectra. **d-g** Visualization of the equilibrated electrolyte systems after 4 ns: **d** 1 m KOAc, **e** 1 m LiOAc, **f** 27 m KOAc, **g** 32 m KOAc-8 m LiOAc. Color scheme: violet: K, green: Li, red: O, grey: C, white: H. Adapted with permission from Ref. [112]. Copyright 2018, The Royal Society of Chemistry

Compared to the first two, research on low-temperature aqueous potassium-ion batteries is relatively limited. Typically, these batteries use dilute base electrolytes such as 0.5 m K_2_SO_4_ and 1 m KCl, due to the high ionic conductivity and low cost. However, dilute electrolytes often lead to hydrolysis, causing short circuits and other issues due to the high activity of water [110]. Although the outlook of the high-concentration salt strategy is not promising, selecting cost-effective inorganic salts can still lead to viable application scenarios. KOAc, inexpensive, non-toxic, and highly soluble, is a viable alternative to toxic imide-based electrolytes, providing an extended electrochemical stability window [111]. Maria et al. [112] evaluated the simultaneous maximum solubility of LiOAc and KOAc in water at different ratios. The eutectic ratio was established at a LiOAc molar fraction of 0.2 when the solubility of the salt was maximized (Fig. 9a). Based on this a lithium-potassium acetate mixture was developed with a water-cation molar ratio as high as 1.3: 32 m KOAc-8 m LiOAc (Li_0.2_K_0.8_OAc∙1.3H_2_O). To characterize the thermal properties of the eutectic and related solutions, differential scanning calorimetry (DSC) was used (Fig. 9b). Differential scanning thermograms of aqueous solutions of 1 m LiOAc, 1 m KOAc, 27 m KOAc, 27 m KOAc-6 m LiOAc, and 32 m KOAc-8 m LiOAc. In contrast, the saturated salt solutions did not show the expected peaks corresponding to the melting transition over the entire range tested, indicating a freezing point below − 60 °C. To investigate the interactions in these highly concentrated solutions, nuclear magnetic resonance (NMR) studies (Fig. 9c), and molecular dynamics (MD) simulations (Fig. 9d-g) were performed, and their behavior was compared to that of the respective 1 m KOAc and LiOAc solutions. Increasing salt concentration in the dual-salt electrolytes results in an upfield shift in the ^7^Li NMR spectrum, which is indicative of ion shielding or increased Lewis basicity in the vicinity of the lithium cation. In highly concentrated electrolytes, the solvation shell of the different ions strongly interpenetrate, and the presence of anions in the coordination sphere of Li^+^ leads to increased electron density on the cation, resulting in the observed upfield peak shift, indicating complexation and pronounced interaction with the anions in solution [113]. The results of MD simulations are consistent with the experimental observations. As the concentration of the double salt solution increases, Indicates a decrease in the amount of free water in solution. It offers a wide electrochemical stability window of 3 V for high concentrations of lithium-potassium acetate mixture. Besides, the “WISE” exhibits electrical conductivity comparable to or higher than typical organic electrolytes.

The use of high-concentration salts and salt additives has emerged as a simple yet effective approach to expanding the low-temperature operational range and electrochemical stability window (ESW). However, several underlying mechanisms remain insufficiently studied and require further systematic investigation: (I) the viscosity of the solution will be increased along with the increase in the concentration, but the specific linkage has yet to be investigated; (II) the effect of the electrode–electrolyte interface of aqueous batteries of the “WISE” system has not been elucidated; (III) the effect of different electrolytes and their concentrations on the electrode material. Furthermore, although “WISE” helps broaden the electrochemical window and form anion-derived interphase layers that suppress water redox reactions, the interphase layers formed in aqueous electrolytes are typically more porous and less stable than those formed in organic electrolytes. Moreover, the issue of slow self-discharge and calendar life degradation caused by the thermodynamic instability of water across wide voltage ranges remains a key bottleneck hindering the practical application of aqueous electrolytes in AMIBs. The investigation of the above issues is of great significance for the design of electrolytes for long-life AAMIBs.

#### Organic Co-solvent Strategy

**Fig. 10:**
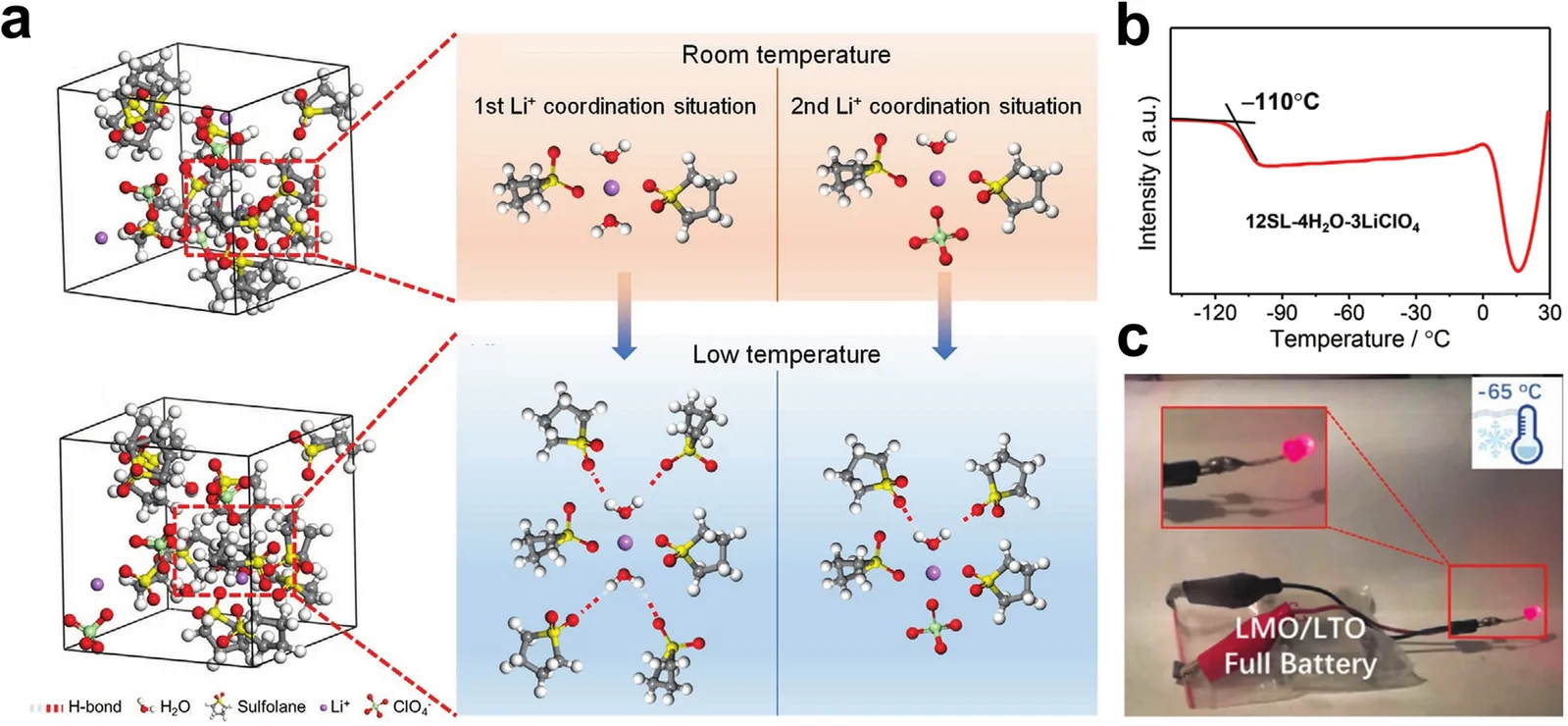
**a** MD simulations of 12SL-4H_2_O-3LiClO_4_ electrolyte at normal and low temperatures. **b** DSC curve of 12SL-4H_2_O-3LiClO_4_ electrolyte. **c** Picture of a working battery at − 65 °C. Adapted with permission from Ref. [114]. Copyright 2021, Wiley–VCH

The salt regulation strategy not only can broaden the electrochemical stability window but also effectively enhance the low-temperature performance of the AAMIBs. However, excessively high salt concentrations often significantly increase the cost of electrolytes. As electrolyte concentration rises, the interaction forces between anions and cations intensify, hindering ion migration and resulting in increased viscosity and decreased conductivity. Furthermore, highly concentrated electrolytes typically face application challenges such as poor electrode wettability and impeded liquid-phase mass transfer, which adversely affect the charging and discharging performance of batteries [114]. These issues restrict their large-scale applications. In contrast, the organic co-solvent strategy can reduce costs while enhancing both fluidity and wettability of the electrolyte without compromising performance by incorporating low-cost organic solvents. Furthermore, incorporating organic solvents into aqueous electrolytes can expand the electrochemical stability window and lower the freezing point of mixed electrolytes [115–117]. The initial reason is that the organic polar solution with a certain characteristic functional group can easily dissolve a variety of inorganic salts and be completely miscible with water. This property helps prevent the formation of hydrogen bond networks among free water molecules at low temperatures, which otherwise leads to freezing. Most organic functional groups are H-bond donors or acceptors, thereby weakening the original H-bonds and inhibiting the freezing of the aqueous solution [118]. Commonly used organic co-solvents are sulfolane (SL), ethylene glycol (EG), ethylene glycol (AN), butyl diacetylene, etc. Taking SL as an example, Li^+^ is preferentially coordinated with water molecules because the donor number of SL (14.8) is smaller than that of water (18). It helps to reduce the proportion of free water in the mixed electrolyte. Liu et al. [114] developed an antifreeze electrolyte: 12SL-4H_2_O-3LiClO_4_. From the MD simulation (Fig. 10a), it can be seen that most of the water molecules are coordinated with Li^+^, in addition to the strong interactions between SL and water molecules, which coordinates most of the water molecules with the salt to form a Li^+^ solvated sheath, and most of the water molecules are immobilized by the anchoring effect of Li^+^, ClO_4_^−^ and SL molecules. During charging and discharging, the immobilized water molecules find it difficult to contact the electrodes compared to [Li(H_2_O)_4_]^+^ and free water molecules in conventional aqueous electrolytes. Therefore, it expands the ESW by changing the solvated structure of Li^+^ ions and reducing the activity of water molecules. In addition, the interaction of SL with water molecules to disrupt the hydrogen-bonding network between water molecules can inhibit the freezing of water. In this way, SL, solvated Li^+^, and water are separated from each other. When the temperature drops below 0 °C, the contact between these components becomes closer, and new bonds appear between SL and water that are coordinated to Li^+^. However, it isn’t easy to form large-scale ordered interactions in 12SL-4H_2_O-3LiClO_4_. As a result, the electrolyte exhibits a disordered structure similar to that of a glassy liquid According to the DSC curve (Fig. 10b), the glass transition temperature of the 12SL-4H_2_O-3LiClO_4_ electrolyte is a very low − 110 °C. As shown in Fig. 10c, the LMO/LTO full cell assembled with the hybrid electrolyte was able to discharge normally and light up the LED at − 65 °C.

**Fig. 11:**
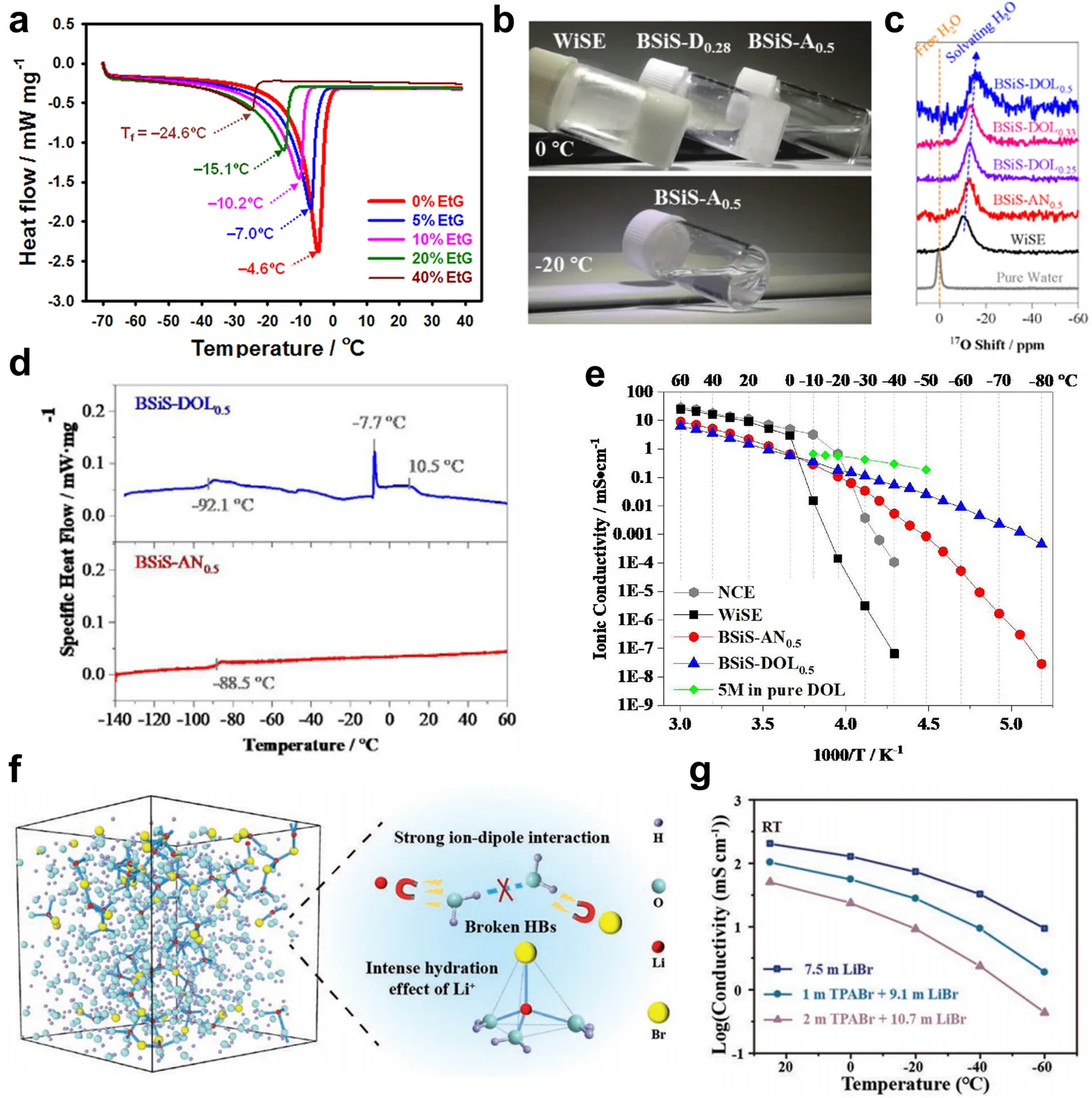
**a** DSC analysis of the aqueous electrolyte solutions with antifreeze additive (EtG, wt %): 0, 5, 10, 20, and 40. Adapted with permission from Ref. [119]. Copyright 2019, American Chemical Society. **b** Photographs showing the states of WISE, BSiS-D_0.28_, and BSiS-A_0.5_ electrolytes at 0 and − 20 °C. Adapted with permission from Ref. [120]. Copyright 2019, Wiley–VCH. **c** ^17^O NMR spectra of electrolytes and water. **d** Ionic conductivity of electrolytes over a wide temperature range. **e** DSC curves of BSiS-AN_0.5_ and BSiS-DOL_0.5_ hybrid electrolytes were collected at a rate of 1 °C min^−1^ from − 140 to 60 °C. Adapted with permission from Ref. [121]. Copyright 2021, Elsevier. **f** Schematic diagram of the H-bonds breakage mechanism in LiBr solution. **g** Conductivities of 7.5 m LiBr and two tailored electrolytes at RT, 0, − 20, − 40, and − 60 °C. Adapted with permission from. Adapted with permission from Ref. [94]. Copyright 2022, Wiley–VCH

Thus, adding organic cosolvent is an effective strategy for improving the low-temperature performance of aqueous alkali metal batteries. EG is a common antifreeze additive used in automotive engines to prevent freezing. Due to its miscibility with water can also be applied to aqueous lithium-ion batteries to enhance low-temperature performance. For example, when a certain amount of EG was added to 1 m Li_2_SO_4_ aqueous solution, the overall ESW expanded and the freezing point of the mixed solution decreased from − 4.6 to − 24.6 °C when the concentration increased from 0% to 40% (Fig. 11a) [119] Similarly, ethyl (AN), an organic co-solvent with a high dielectric constant (35.9), low freezing point (− 48 °C), high oxidation stability, and high miscibility with water, is also very suitable for improving the low-temperature performance of aqueous batteries. Chen et al. [120] demonstrated that combining AN with the aqueous electrolyte LiTFSI in ultra-high-concentration (15.3 M) salt significantly enhanced the ionic conductivity of the mixed solution. This addition also facilitated the interaction between H_2_O molecules and Li^+^, thus reducing the reactivity of water and maintaining the mixed electrolyte in a liquid state at − 20 °C (Fig. 11b). A wide ESW of 4.5 V was achieved due to new interphase chemical reaction and the exclusion of water molecules by TFSI^−^ and AN molecules on the anode surface.

Despite these advancements, the low-temperature performance of aqueous electrolytes remains suboptimal. Ma et al. [121] proposed using 1, 3-dioxane (DOL) as a cosolvent due to its high reduction stability, low viscosity, and extremely low freezing point (− 95 °C). Although DOL is flammable, the high water content in the mixed electrolyte makes it non-combustible. The addition of DOL to the WISE shifts the ^17^O NMR signal upward, indicating a negative chemical shift (Fig. 11c). This shift suggests that the interaction between Li^+^ and water molecules in BSiS-DOL_0.5_ is stronger than that in other systems, shielding oxygen nuclei in water. This interaction reduces the number of free water molecules on the anode surface, thereby expanding the electrochemical stability window to 4.7 V. Due to the reduction of free water content, which also lowers the freezing point of the electrolyte, differential scanning calorimetry (DSC) analysis shows that the glass transition of BSiS-DOL_0.5_ occurs at − 92.1 °C (Fig. 11d). In addition, the ionic conductivity of the electrolyte is significantly higher than that of the WISE solution at low temperatures (Fig. 11e). However, aqueous electrolytes containing organic cosolvents often suffer from low ionic conductivity, especially at very low temperatures. To address this, Wang et al. [94] designed an ultra-low temperature high-performance aqueous lithium-ion bromine battery (ALBB) with lithium bromide (LiBr) and tetrapropylammonium bromide (TPABr) as electrolytes (Fig. 11f). The strong ionic dipole interaction between Br^−^ and H_2_O combined with the robust hydration effect of Li^+^, enables the battery to perform well at low temperature, maintaining high conductivity (1.89 mS cm^−1^) at − 60 °C (Fig. 11g). Furthermore, the excellent bromine fixing properties of TPABr and the rapid redox reaction of Br_2_/Br^−^ endow the battery with high reversible cathode capacity and excellent rate performance at low temperatures, achieving a capacity retention rate of up to 98% at − 60 °C.

**Fig. 12:**
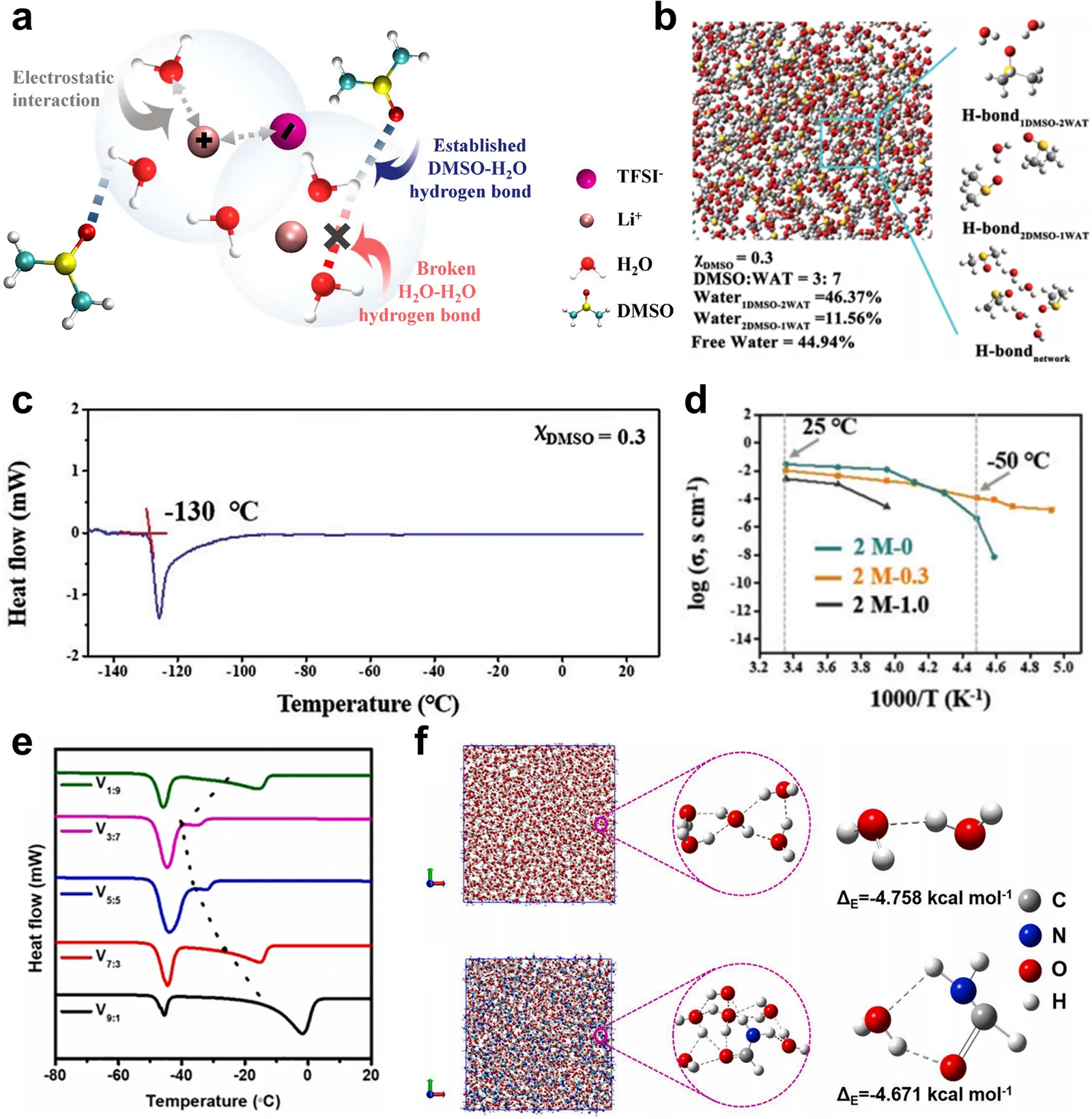
**a** Schematic illustration of the possible supramolecular interactions among LiTFSI, DMSO, and water. Adapted with permission from Ref. [123]. Copyright 2022, Elsevier. **b** Conformation analysis of the system with X_DMSO_ = 0.3 from MD simulations. **c** DSC for X_DMSO_ = 0.3 electrolyte solvent. **d** Temperature-dependent ionic conductivity study of solutions with different amounts of DMSO added. Adapted with permission from Ref. [125]. Copyright 2019, Wiley–VCH. **e** DSC results of various solutions with different volume ratios between H_2_O and FA with a heating rate of 5 °C min^−1^. **f** Optimized structure of the pure water from MD simulations and the intermolecular interaction among water molecules from DFT calculations; The optimized structure of the optimized solution from MD simulations and the intermolecular interaction among H_2_O-FA clusters from DFT calculations. Adapted with permission from Ref. [126]. Copyright 2022, Elsevier

Dimethyl sulfoxide (DMSO) is another highly polar, sulfur-containing compound with a high boiling point, capable of dissolving most inorganic salts and being fully miscible with water. As the H-bond acceptor, DMSO has been a preservative and freezing medium for biological tissue since 1959 [122]. DMSO weakens the interaction of water with cations. Still, the O atoms in DMSO can also form strong H-bonds with the H atoms in H_2_O. This can significantly reduce the electron density of hydrogen atoms in H_2_O, lowering the energy level and stabilizing the hydrogen atoms [123] (Fig. 12a). In early research, Havemeyer discovered that the H_2_O/DMSO mixture (with a DMSO molar fraction of 0.30) exhibited an extremely low freezing temperature [124]. Nian et al. [125] found through simulation studies that the X_DMSO_ = 0.30 system formed DMSO-H_2_O H-bonds reached a fairly stable state, and most of its DMSO molecules were in “1DMSO-2H_2_O” aggregates. The conformational analysis of this particular system was performed, and the results are shown in Fig. 12b. This property was utilized to develop an electrolyte system for aqueous sodium-ion batteries using a binary solution of H_2_O/DMSO (X_DMSO_ = 0.30) 2 M NaClO_4_ with a solution concentration of 2 M-0.3. This system maintains an ultra-low freezing point below − 130 °C. It retrains a high ionic conductivity of 0.11 mS cm^−1^ at − 50 °C (Fig. 12c, d). This is because by increasing the concentration of DMSO, strong H-bonds are formed between the H of the O–H bonds in the water molecules and the O of the S=O bonds, and the H-bond interactions between the water molecules are weakened. This produces an electrolyte with excellent low-temperature properties. Similarly, Zhu et al. [126] introduced formamide (FA), a strong polar solvent, as a cosolvent in a 17 m NaClO_4_. The optimized electrolyte achieved a minimum freezing point below − 50 °C (Fig. 12e) and could provide 8000 cycle life at − 50 °C and 4 C. Theoretical calculations suggest that the average interaction energy between water and FA is comparable to that between water molecules, leading to the spontaneous formation of H₂O-FA clusters through intermolecular H-bonds (Fig. 12f). The carbonyl and amino groups of FA coordinate with hydroxyl groups in water, disrupting the hydrogen bond network and preventing the formation of long-range ordered structures at sub-zero temperatures, thus further reducing the freezing point [127]. Additionally, glycerol (Gly) also disrupts the hydrogen bond network in water, expanding the electrochemical stability window. Sun et al. [128] designed dilute electrolytes using Gly and 1 M NaNO_3,_ achieving a low freezing point below − 80 °C and a wide electrochemical stability window of 2.7 V.

Additionally, potassium hydrate ions have a smaller radius than lithium and sodium hydrate ions, which means they have higher conductivity and ion transport rates in aqueous solutions, indicating a promising future for aqueous potassium-ion batteries [129]. However, research on them remains limited. Introducing an organic cosolvent into an aqueous electrolyte is an effective strategy to increase salt concentration relative to the water component while also controlling the interfacial chemistry. While organic co-solvent strategies offer advantages in enhancing battery performance, certain organic solvents may be costly or environmentally detrimental, and some co-solvents can exhibit flammability or toxicity, posing safety risks in battery applications and thereby limiting their feasibility for large-scale applications [130]. It is crucial for practical applications to explore more environmentally friendly and safe additives. In contrast, hydrogel electrolytes demonstrate superior environmental compatibility and biodegradability, while their high water content contributes to enhancing battery safety. Moreover, the excellent mechanical stability and high ion conductivity of hydrogel electrolytes also contribute to improving the structural stability and electrochemical performance of batteries. Therefore, this strategy complies with the current technical requirements and holds greater development potential.

#### Hydrogel Strategy

**Fig. 13:**
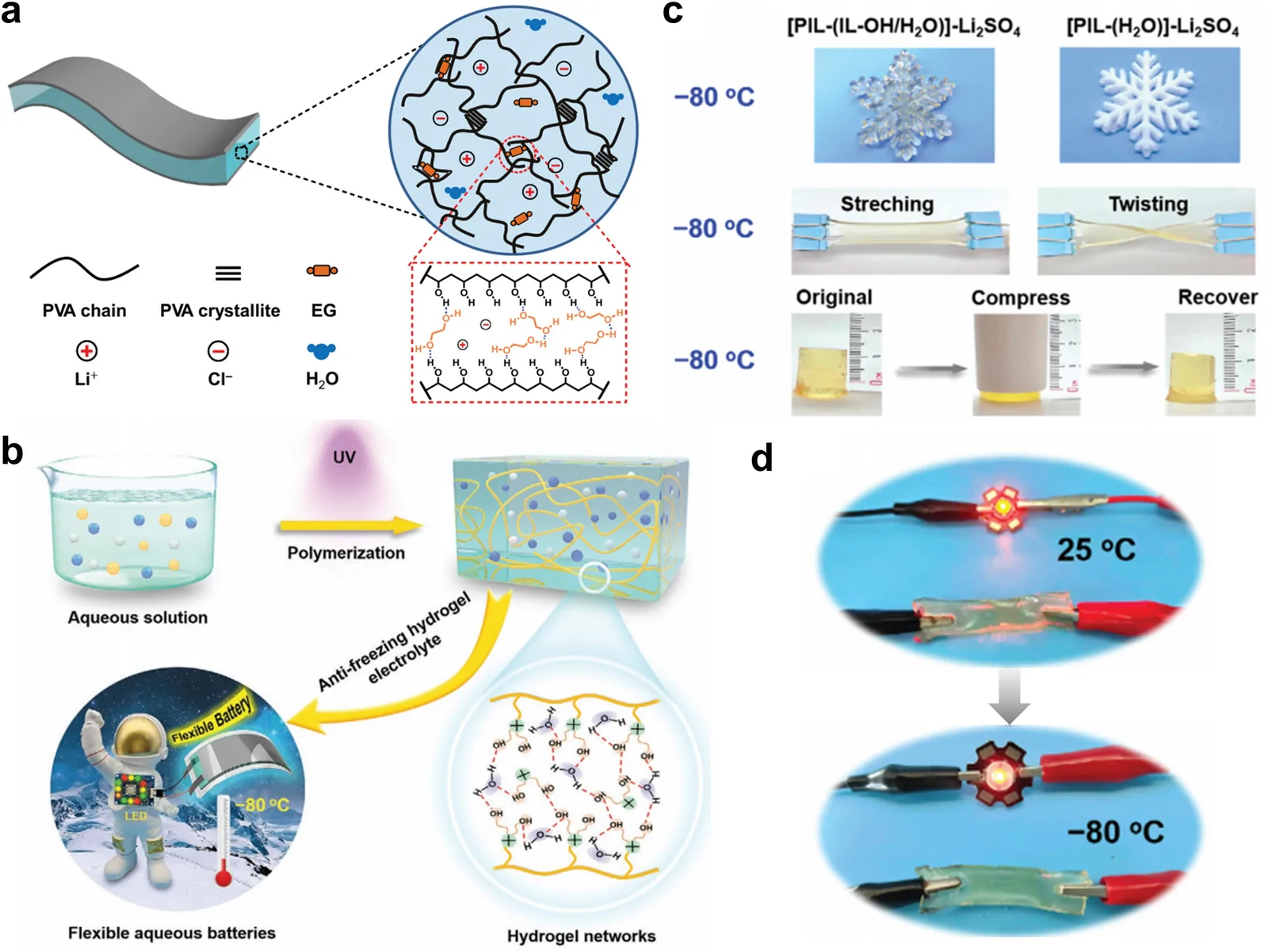
**a** Schematic illustration of the organic hydrogel electrolyte applied on carbon nanotube paper electrodes. Adapted with permission from Ref. [73]. Copyright 2018, Wiley–VCH. **b** Preparation and components of the anti-freezing PIL-OH hydrogel. Taking advantage of the PIL-OH hydrogel, the flexible aqueous lithium-ion cell showed outstanding low-temperature performance and can be operated in environments as low as − 80 °C. **c** Optical photograph of [PIL-(IL-OH/H_2_O)]-Li_2_SO_4_ and [PIL-(H_2_O)]-Li_2_SO_4_ hydrogels, and the mechanical properties of the [PIL-(IL-OH/H_2_O)]-Li_2_SO_4_ hydrogel at − 80 °C. **d** [PIL-(IL-OH/H_2_O)]-Li_2_SO_4_ hydrogel applied as an ionic conductor, which could light an LED bulb at − 80 °C. Adapted with permission from Ref. [136]. Copyright 2022, Wiley–VCH

Hydrogels are functional materials known for their superabsorbent properties, characterized by a cross-linked polymer network that allows for substantial water adsorption and retention. Due to these properties, hydrogels can significantly enhance the low-temperature performance of AAMIBs. Their excellent water absorption helps maintain electrolyte fluidity at low temperatures, while their water retention capabilities prevent water in the electrolyte from freezing or evaporation. Additionally, hydrogels can provide pathways for electron and ion transport, contributing to high conductivity at room temperature, and making them suitable for energy storage devices [131–133]. However, at extremely low temperatures, the water within hydrogels can freeze. To address this issue, researchers have explored various strategies, such as adding lipophilic components into the polymer network during the polymerization process [87, 134] or adding high-concentration solute to hydrogels to lower the freezing point of the mixed solution [135]. Although these methods can reduce the freezing point, they do not necessarily improve ionic conductivity at low temperatures. Therefore, it is necessary to develop hydrogel electrolyte materials that combine high ionic conductivity with resistance to low temperatures. Inspired by fat species in natural biological organisms, Rong et al. [73] investigated a novel organic hydrogel electrolyte consisting of hydrogen-bonded cross-linked PVA networks and PVA crystals. Figure 13a shows the anti-freezing organic hydrogel electrolyte applied to carbon nanotube paper electrodes. In the organic hydrogel electrolyte, PVA can be swollen by the solvent and crosslinked with the solvent molecules, and the EG molecules can form a large number of H-bonds with the PVA chains, inducing the generation of PVA crystal structure domains, therefore lowering the freezing point of the gel electrolyte. In addition, the H_2_O/EG binary solvent contained in the PVA gel can also dissolve ions, which makes the PVA gel a good ionic conductor even at low temperatures. In addition, the agent molecules can form H-bonds with the PVA chains, which greatly improves the mechanical strength of the organic hydrogel, and the organic hydrogel electrolyte maintains excellent flexibility even when the temperature drops to − 40 °C, compared to the conventional hydrogel electrolyte that breaks easily when flexed at − 20 °C. To further broaden the temperature range for hydrogel electrolyte applications, poly (ionic liquids)s (PILs), which combine the characteristics of green ionic liquid (IL) and polymer framework, have attracted increasing interest. Hu et al. [136] prepared a hydroxyl-functionalized poly (ionic liquid)-based (PIL-OH)-hydrogel electrolyte with PIL-OH as the backbone and IL-OH/H_2_O as the dispersion medium. Figure 13b shows the synthesis process of PIL-OH hydrogel. The polymer chains of PIL-OH form H-bonds with IL-OH and H_2_O to form a non-covalently cross-linked IL-OH hydrogel. Among them, IL-OH with ionic centers and hydroxyl substituents can interact with water molecules through H-bonds and ionic hydration, which improves the antifreeze properties. Figure 13c shows an optical photograph of PIL-OH hydrogel at − 80 °C. The [PIL-(IL-OH/H_2_O)]-Li_2_SO_4_ hydrogel does not crystallize, indicating that it has better antifreeze properties. In addition, the hydrogel electrolyte maintained reversible tensile and torsional properties at − 80 °C. The hydrogel was further connected to the LED light circuit to light up the LED (Fig. 13d).

**Fig. 14:**
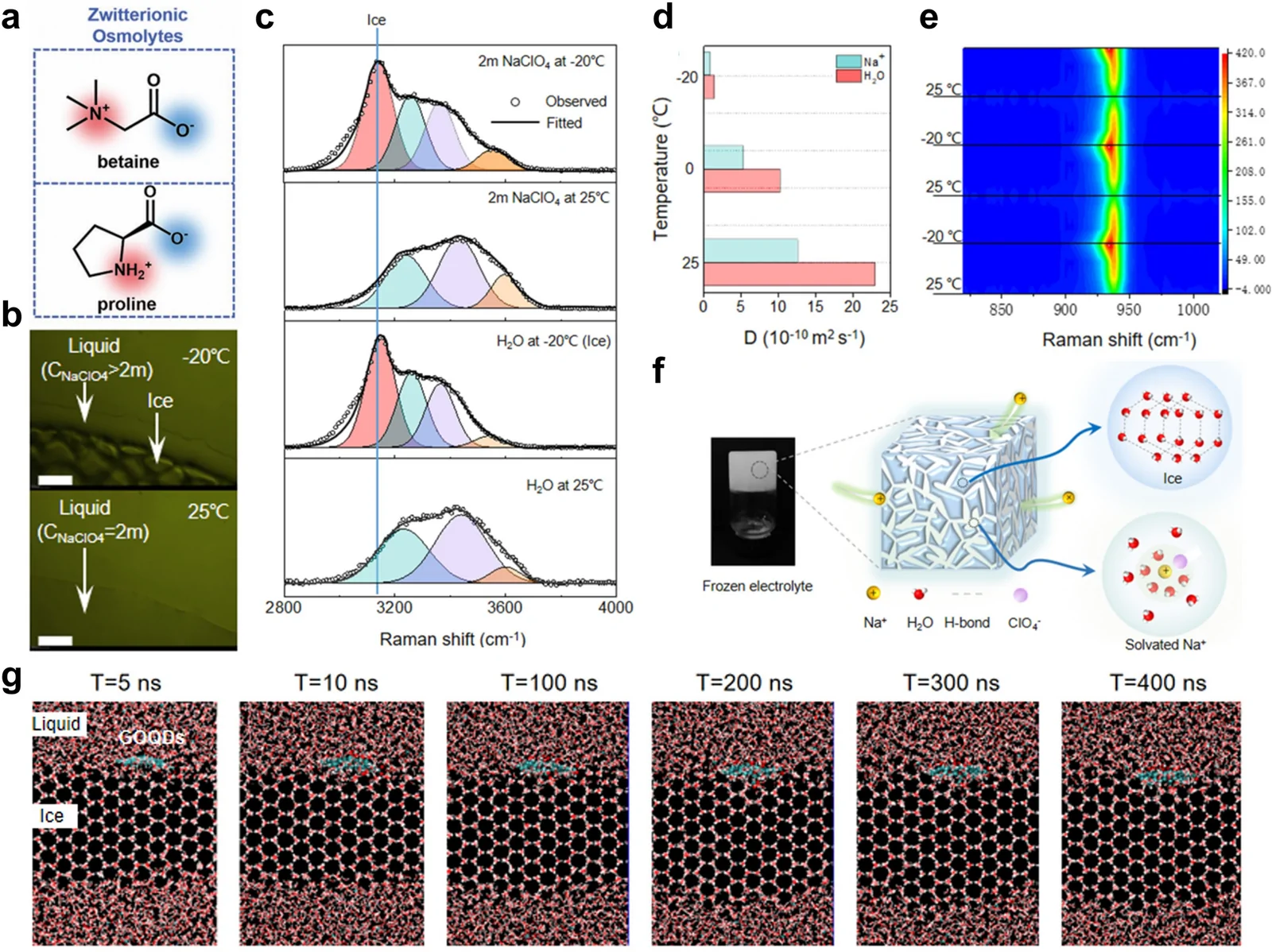
**a** Molecular structures of betaine and proline. Adapted with permission from Ref. [133]. Copyright 2019, Wiley–VCH. **b** Microscope observation of 2 m NaClO_4_ electrolytes at 25 °C and − 20 °C. The scale bar is 200 μm. **c** O–H stretching vibration of H_2_O and 2 m NaClO_4_ electrolytes at 25 °C and − 20 °C. **d** Na^+^ and H_2_O self-diffusion coefficients for the 2 m NaClO_4_ electrolytes from pfg NMR experiments at 25, 0, and − 20 °C. **e** Contour map of Raman spectrum of 2 m NaClO_4_ electrolyte during cooling and heating. **f** The schematic of the structure of 2 m NaClO_4_ electrolytes at − 20 °C. **g** Snapshots of the effect of GOQDs on the ice crystal growth simulation at each time (5, 10, 100, 200, 300, and 400 ns) at − 30 °C. Adapted with permission from Ref. [137]. Copyright 2023, Wiley–VCH

Zwitterions are ionic compounds containing both acidic and basic functional groups. Acidic groups are typically negatively charged (e.g., − COOH, –PO_4_H_2_), while basic groups carry a positive charge (e.g., –NH_2_), making these compounds electrically neutral in solution. For instance, certain plants produce betaine or proline during winter to enhance cold tolerance (Fig. 14a). Building on this concept, Sui et al. [133] introduced a calcium alginate/polyacrylamide (PAAm) hydrogel containing NH_4_Cl. This modified hydrogel exhibited a high ionic conductivity of 27 mS cm^−1^ at − 40 °C. The presence of zwitterions improved the ionic conductivity at low temperatures by forming ion pairs in the electrolyte, thereby increasing the total ion concentration. This increase in ion concentration enhances the ionic conductivity of the solution. Additionally, zwitterions can create larger ionic aggregates or dispersions in solution with higher electrical conductivity, accelerating ion transport. Nian et al. [137] revealed the general phase evolution principles and potential ionic conduction mechanisms of frozen aqueous electrolytes. Inspired by the knowledge of cellular cryopreservation based on biological research, the design of a colloidal dispersed electrolyte containing graphene oxide quantum dots (GOQDs) efficiently inhibited the growth of ice crystals. It enlarged the interconnecting liquid region for ion transport. They first studied the freezing properties of four water electrolytes and found that the electrolytes still worked after freezing at temperatures below freezing, suggesting that it is a common phenomenon that frozen water electrolytes can conduct ions. To understand the general ionic conduction mechanism in frozen electrolytes, the solvated structure of 2 m NaClO_4_ electrolytes at different temperatures was investigated. Micrographs of the electrolyte before and after freezing are shown in Fig. 14b, where the electrolyte interface still contains a large amount of liquid, and in situ variable-temperature Raman results confirm the presence of liquid regions in the frozen electrolyte (Fig. 14c). For pure water, at 25 °C, the main peak is located at ~ 3440 cm^−1^ and the water molecules are mainly in the form of double donor-single acceptor H-bonds (DDA). At − 20 °C, the sharp peak at ~ 3150 cm^−1^ is the tetrahedral H-bonds (DDAA) structure of the H_2_O molecule, which is the dominant form of H-bonds in ice. Dispersed solutes may be present in the water channels remaining between the ice crystals, and in-situ variable-temperature pulsed-field-gradient (pfg) NMR results (Fig. 14d) show that even if the electrolyte is frozen, there is still diffusion of water molecules due to the presence of liquid regions on the surface of the ice crystals. The in-situ variable-temperature Raman (ivt-Raman) results in Fig. 14e show the reversible evolution of the electrolyte structure during cooling and heating. Figure 14f shows a detailed schematic of ion transport in a frozen electrolyte, where ions are conducted along interconnected liquid regions. Based on these findings, inspired by the fact that the introduction of solid particles can regulate the growth of ice crystals in saline and expand the liquid phase range of the frozen solution for cellular cryopreservation [137–140], graphene oxide quantum dots (GOQDs) with abundant oxygen-containing groups are introduced to exploit their interaction with potential ice crystals. To investigate the microscopic interactions between GOQDs and frozen electrolytes, molecular dynamics (MD) simulations were performed at − 30 °C to analyze the growth of ice crystals in a 2 m NaClO_4_ electrolyte with or without GOQDs (Fig. 14g). It was found that ice crystals grew layer by layer on the side of the ice plate without GOQDs, while the side with GOQDs significantly inhibited the growth of ice crystals, and further growth could only take place on its two sides. The designed 2 m NaClO_4_ + 1 mg mL^−1^ GOQDs colloidal dispersed electrolyte has higher ionic conductivity and a wider ESW, and the assembled aqueous cell has superior low-temperature performance. This novel colloidal electrolyte design strategy demonstrates the feasibility of “unconventional” electrolyte additives for battery applications. Table 1 summarizes key examples of electrolyte innovations in low-temperature aqueous alkali metal ion batteries.

**Table 1:** Low-temperature aqueous alkali metal ion batteries realized by electrolyte design

Strategy		Cathode//Anode	Electrolyte	Freezing point (°C)	Ionic conductivity (mS cm^−1^)@°C	Capacity retention (%)@T(°C)	References
Highly concentrated salt	Li	LiMn_2_O_4_//Mo_6_S_8_	21 m LiTFSI	–	–	–	[97]
		Li_3_V_2_(PO_4_)_3_//LiTi_2_(PO_4_)_3_	21 M LiTFSI	− 20	–	60@ − 20	[100]
		LiMn_2_O_4_//AC	5.2 m LiTFSI	− 43.4	1.8@ − 40	66.4@ − 40	[101]
		LiCoO_2_	16 m LiCl	− 45	–	72@ − 40	[102]
		LiCoO_2_	9.5 m LiNO_3_	− 30	–	–	[102]
		LiCoO_2_	3.5 m Li_2_SO_4_	− 35	–	–	[102]
	Na	Ni(OH)_2_//NaTi_2_(PO_4_)_3_@C	2 M NaClO_4_	− 20	–	85@ − 20	[105]
		NiHCF//PT	17 M NaClO_4_	− 40	4.4@ − 40	68.7@ − 40	[103]
		Na_3_V_2_(PO_4_)_3_//Na_3_V_2_(PO_4_)_3_	17 M NaClO_4_	− 40	4.4@ − 40	88@ − 40	[106]
		Na_2_MnFe(CN)_6_//NaTi_2_(PO_4_)_3_/C	32 m KAc + 8 m NaAc	–	12@25	–	[108]
		Na_2_CoFe(CN)_6_//AC	1 m NaClO_4_ + 3.86 m CaCl_2_	− 100	7.13@ − 50	64.8@ − 30	[39]
		Na_2_MnFe(CN)_6_//NaTi_2_(PO_4_)_3_/C	17 M NaClO_4_	− 40	4.4@ − 40	–	[96]
		NaFeMnHCF//NaTi_2_(PO_4_)_3_	1 m NaCF_3_SO_3_ + 2.5 m Al(CF_3_SO_3_)	− 86.1	–	–	[109]
		NaFeMnHCF//NaTi_2_(PO_4_)_3_	1 m NaClO_4_ + 4 m Ca(ClO_4_)_2_	− 117.1	–	–	[109]
		NaFeMnHCF//NaTi_2_(PO_4_)_3_	H_50_EG_50_-2 m NaCF_3_SO_3_	− 114.5	–	–	[109]
		Na_2_CoFe(CN)_6_//AC	0.5 m NaCl + 4 m MnCl_2_	− 50	2.44@ − 50	54.9@ − 40	[141]
	K	c-TiO_2_//LiFePO_4_	32 m KAc + 8 m LiAc	− 40	5.3@25	90@25	[112]
		K_1.85_Fe_0.33_Mn_0.67_[Fe(CN)_6_]_0.98_·0.77H_2_O//PTCDI	22 M KCF_3_SO_3_	− 20	10@ − 20	76.3@ − 20	[142]
Co-solvent	Li	LiFePO_4_	EG-Li_2_SO_4_	− 24.6	4.25@ − 20	82@ − 20	[119]
		LiMn_2_O_4_//Li_4_Ti_5_O_12_	AN-LiTFSI	− 20	0.63@ − 20	95@0	[119]
		LiMn_2_O_4_//Li_4_Ti_5_O_12_	DOL-LiTFSI	− 92.1	7.09@60	52@ − 50	[121]
		LiMn_2_O_4_//Li_4_Ti_5_O_12_	SL-LiClO_4_	− 110	0.05@ − 50	98@ − 20	[114]
		PC//NDPI	TPABr + LiBr	− 60	1.89@ − 60	98@ − 40	[94]
	Na	NaTi_2_(PO_4_)_3_//AC	DMSO + NaClO_4_	− 130	0.11@ − 50	60@ − 50	[125]
		PNTCDA//AC	FA + NaClO_4_	− 50	1.75@ − 50	52.5@ − 50	[126]
		Ni_2_ZnHCF//PTCDI	Gly + NaNO_3_	− 80	–	80@ − 10	[128]
	K	CuHCF//p-PTCDI-EDA	HBCD + KCF_3_SO_3_	− 20	–	97.8@10	[143]
Hydrogels	Li	LiTi_2_(PO_4_)_3_//AC	PIL-OH	− 80	0.08@ − 80	36@ − 80	[136]
	Na	NaTi_2_(PO_4_)_3_//AC	NaClO_4_ + GOQDs	− 30	–	74@ − 30	[137]
		Na_2/3_Mn_2/3_Co_1/3_O_1.98_F_0.02_//HC	2 m NaTFSI + 10 wt% H_2_O	− 25	–	30@ − 25	[144]
	K	PPy//K	KPF_6_ + PC/FEC	− 10	–	–	[145]

### High-Temperature Design Strategy

Compared to the extensive research on improving the low-temperature tolerance of aqueous electrolytes, studies focusing on enhancing their high-temperature tolerance are relatively limited. In aqueous battery electrolyte research, both low- and high-temperature modification strategies are employed to enhance battery performance by optimizing the electrolyte’s physical and chemical properties. The low-temperature modification strategy primarily aims to improve ionic conductivity and reduce free water content, mitigating the issue of sluggish ion transport at low temperatures. Conversely, the high-temperature modification strategy focuses on enhancing the electrolyte’s thermal stability and minimizing side reactions at elevated temperatures. This approach helps prevent electrolyte decomposition and thermal runaway. Two major challenges associated with operating aqueous batteries at high temperatures include electrolyte evaporation and electrode capacity degradation. Similar to organic solvent-based batteries, the performance of aqueous batteries is influenced by the physical and chemical properties of water at high temperatures. The strength of the hydrogen bond network directly affects interactions between water molecules, thereby influencing water evaporation behavior. As temperature increases, intensified molecular motion weakens H-bond interactions, leading to accelerated water evaporation and reduced adaptability of aqueous batteries to high-temperature conditions [146, 147]. Additionally, elevated temperatures increase water molecule activity and reduce the overpotential for water electrolysis, thereby accelerating the kinetics of the hydrogen evolution reaction (HER) and oxygen evolution reaction (OER). The gas bubbles generated during these processes can block electrode surfaces, further hindering electrochemical reactions [148]. Therefore, enhancing the “water retention” capability of aqueous electrolytes at high temperatures is a crucial research direction.

**Fig. 15:**
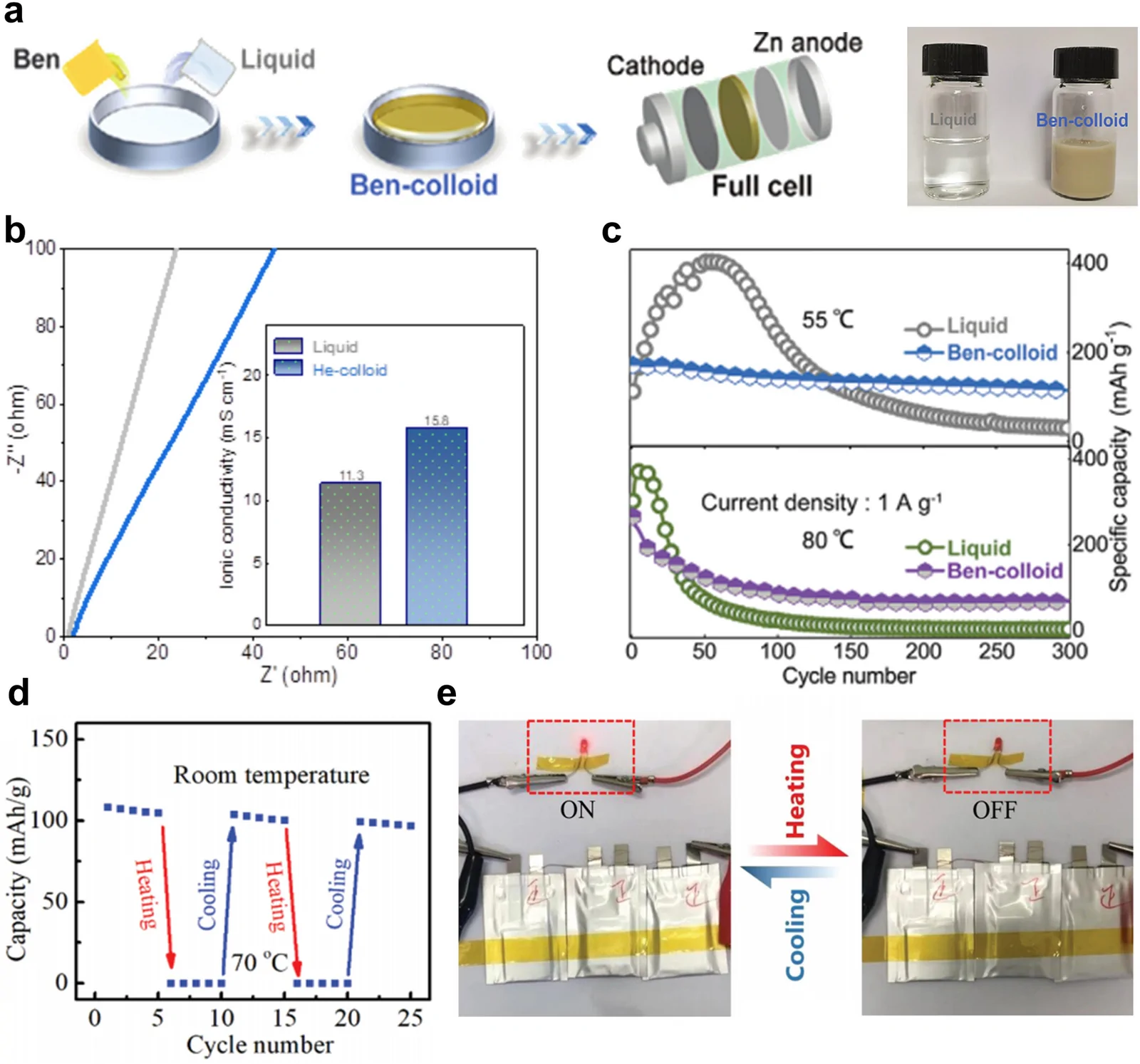
**a** Schematic synthesis of Ben-colloid electrolyte and Ben-colloid electrolyte compared with liquid electrolyte. **b** AC impedance curves and their corresponding ionic conductivity (inset) in liquid and Ben-colloid electrolytes, respectively. **c** Electrochemical performance of Zn/MnO_2_ batteries for cyclic at 55 and 80 °C. Adapted with permission from Ref. [149]. Copyright 2021, Wiley–VCH. **d** Self-protection in several heating–cooling cycles. **e** Picture of conversion of LED lighting and shut off. Adapted with permission from Ref. [156]. Copyright 2022, Wiley–VCH

Adding colloidal electrolytes is a promising method for enhancing the high-temperature performance of aqueous batteries. At elevated temperatures, the polarization phenomenon of the battery will increase, resulting in faster electrolyte consumption, and then reducing the coulomb efficiency. Xie et al. [149] addressed this issue by developing a high-temperature resistant “Ben-colloid” colloidal electrolyte, incorporating inorganic clay bentonite. This colloidal electrolyte improved the high-temperature performance. The system electrolyte maintains excellent colloidal properties over a long period (5 days) (Fig. 15a). Moreover, the “Ben-colloid” electrolyte exhibited higher ionic conductivity (15.8 mS cm^−1^) compared to the liquid electrolyte (11.3 mS cm^−1^) (Fig. 15b). This mixed colloid electrolyte demonstrated good high-temperature durability (up to 80 °C) maintaining a capacity of 114.9 mAh g^−1^ after 300 cycles at 55 °C and a current density of 2 A g^−1^ (Fig. 15c). This indicates that colloidal electrolyte can significantly enhance the high-temperature performance of aqueous batteries. A strategy that could also be applied to improve the high-temperature performance of other AAMIBs. Additionally, there has been considerable research on improving high-temperature performance in organic electrolyte systems. For example, some researchers have used the anion receptor TPFPB as an electrolyte additive. The strong interaction between the anion and the electron-deficient boron atom in TPFPB weakens the solvation of ClO_4_^−^, enhances the coordination ability between the solvent and Na^+^, and significantly improves the oxidation stability. This approach enables sodium-ion batteries to maintain good cycle performance at a high temperature of 60 °C [150]. These findings offer new directions for further exploration of high-temperature AAMIBs.

At high temperatures, the biggest safety issue facing batteries is emergency overheating, although the high-temperature performance of aqueous batteries can be improved by adjusting the electrolyte components to improve water retention, the design of a high-temperature self-protecting electrolyte material will be more conducive to improving the safety of batteries [151, 152]. One promising approach is using colloidal electrolytes to enhance the high-temperature performance of aqueous batteries. Poly(N-isopropyl acrylamide) (PNIPAm)-based hydrogels are intelligent, thermally responsive materials widely used in self-healing materials and drug delivery systems. These hydrogels exhibit a unique property: above a certain temperature, their polymer chains switch from a hydrophilic state to a hydrophobic state, which is reversible when the temperature drops below a critical threshold [153–155]. Leveraging this property, Yang et al. [156] designed a self-protecting aqueous lithium-ion battery that automatically blocks ion transport channels at high temperatures and resumes normal function upon cooling. The temperature at which this blocking occurs can be adjusted between 30 and 80 °C by modifying the ratio of hydrophilic groups and NIPAm in the hydrogel. This smart battery exhibits a specific capacity near zero at 70 °C but achieves a specific capacity of 110 mAh g^−1^ at room temperature with the rate of 1 C, and demonstrates good cycling performance (Fig. 15d). To further demonstrate self-protection behavior in the battery pack, three pocket cells are connected in series to power a light-emitting diode (LED) (Fig. 15e). When the temperature rises to 70 °C, the LED is automatically extinguished and relit upon cooling to room temperature, indicating the self-protection ability of the smart separator and reversibility. However, while this intelligent material provides thermal protection by shutting down at high temperatures, it does not enhance the overall high-temperature performance of the aqueous battery. Therefore, the rational combination of high-temperature-resistant electrolyte materials and such self-protecting materials is a good research angle.

Based on the above analysis, the core pain points of high temperatures are the acceleration of side reactions and interface instability. Existing strategies often fail to strike a balance between the two aspects. The subsequent research approach can shift from the electrolyte’s “passively enduring” high temperatures to “actively responding” to them. On the one hand, the solvation structure of the electrolyte requires further optimization. The activity of water is the root cause of high-temperature side reactions. In addition to using high-concentration salt to lock in water, some specific polymers or cyclic molecules can also be added to the electrolyte. These molecules can fix water molecules more effectively at high temperatures through stronger hydrogen bonding, thereby reducing their activity. On the other hand, some special materials can also be sought to serve as electrolyte additives. They do not affect conductivity at room temperature but are activated at high temperatures to form an electrode protective layer and even act as a repair agent.

## Wide-Temperature Electrolyte Design Strategy for AAMIBs

At present, there is an increasing demand for batteries for all-climate operations, particularly in applications like grid energy storage and electric vehicles. However, due to the limitations of the high freezing point and low boiling point of the hydrolysate [157], there are limited strategies available to regulate electrolyte performance across a wide temperature range in aqueous batteries. The transition from isolated low- or high-temperature modification strategies to a comprehensive wide-temperature range strategy is an emerging trend in current research. This approach aims to optimize electrolyte performance across a broad temperature range by addressing both low- and high-temperature challenges. When designing electrolytes, researchers must simultaneously improve ionic conductivity and reduce viscosity at low temperatures while ensuring electrolyte stability at high temperatures. A key aspect of the wide-temperature range strategy involves fine-tuning the solvation structure of the electrolyte to facilitate efficient ion transport under varying thermal conditions. Fundamentally, improving the water retention capacity of electrolyte materials is essential. Increasing salt concentration and incorporating organic co-solvents can regulate H-bonds in aqueous solutions, lower water activity, and enhance electrolyte thermal stability. Moreover, these strategies contribute to the formation of a stable SEI layer, which protects the electrode. Hydrogel materials, composed of hydrophilic polymers with a three-dimensional network structure, can effectively trap and retain significant amounts of water. Their stability and mechanical properties can be enhanced through physical and chemical cross-linking. Additionally, their environmental responsiveness and biocompatibility enable them to absorb and retain water efficiently. These properties make hydrogels highly effective in water retention applications, significantly improving the wide-temperature performance of AAMIBs.

Further exploration of electrode materials and fluid-gathering materials is necessary to ensure compatibility with electrolytes under extreme temperatures. Electrolytes can stabilize electrode materials through careful molecular design [158]. Liang et al. [103] introduced Na_4_Fe(CN)_6_ into the electrolyte, which effectively suppressed Mn dissolution in Prussian blue cathode materials, leading to enhanced cycling stability. Two primary research directions for aqueous batteries involve the surface modification of metal-based collectors and the development of novel non-metallic collectors [159, 160]. The fundamental goal is to establish a stable electrode/electrolyte interface while minimizing side reactions such as corrosion and hydrogen evolution. Moreover, artificial intelligence (AI) can facilitate the design of wide-temperature electrolytes by enabling rapid screening of molecules with targeted properties, thereby accelerating electrolyte development [161, 162]. These material design advancements, closely linked to electrolyte optimization, present new opportunities for the future development of wide-temperature AAMIB technology.

### High-Concentration Salt Strategy

**Fig. 16:**
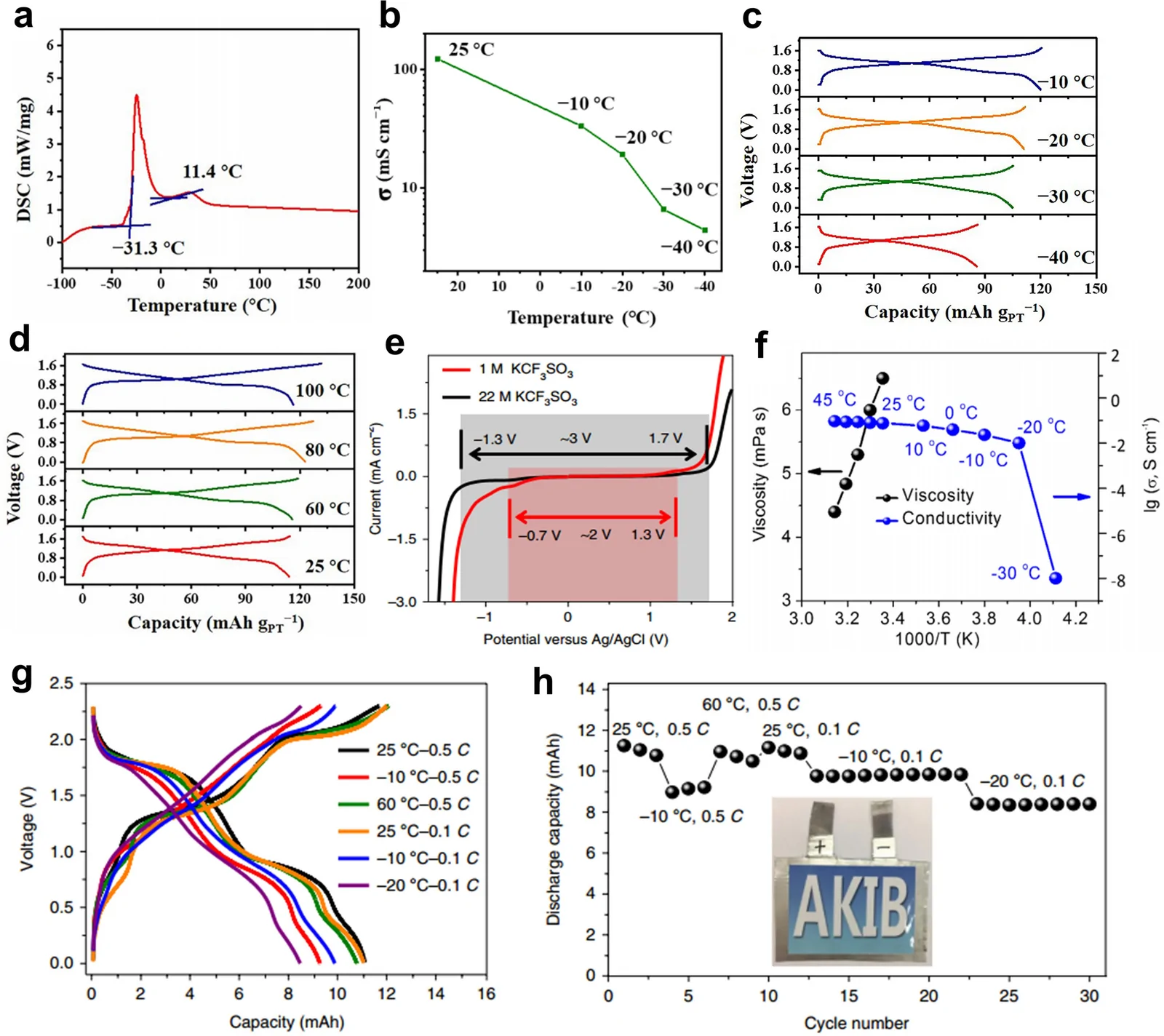
**a** DSC measurement of the 17 m NaClO_4_ electrolyte. **b** Calculated temperature-dependent ionic conductivities of 17 m NaClO_4_ electrolyte. **c** Galvanostatic discharge–charge curves of the NiHCF//PT full cell from − 10 to − 40 °C at 5 A g_PT_^−1^ and **d** from 25 to 100 °C at 0.5 A g_PT_^−1^. Adapted with permission from Ref. [165]. Copyright 2021, Elsevier. **e** Linear sweep voltammetry profiles were recorded on titanium mesh at 10 mV s^−1^ in 1 M and 22 M KCF_3_SO_3_ electrolytes. The electrochemical stability windows of the 1 M and 22 M KCF_3_SO_3_ electrolytes are marked red and black, respectively. **f** The viscosity and conductivity at different temperatures for the 22 M KCF_3_SO_3_ electrolyte. **g** Performance of the KFeMnHC//22 M KCF_3_SO_3_//PTCDI pouch cell at different rates (0.5/0.1 C) and temperatures (− 20/ − 10/25/60 °C) from 0 to 2.3 V for typical charge–discharge curves and **h** the cycling performance. The inset of **e** displays an optical photograph of the assembled aqueous potassium-ion battery pouch cell. Adapted with permission from Ref. [142]. Copyright 2019, Springer Nature

In recent reports on aqueous sodium-ion batteries, the repeated insertion/removal of Na^+^ in electrode material often leads to structural collapse, resulting in limited cycle stability. Carbonyl organic compounds with wide internal space can effectively buffer volume changes [163, 164]. Based on the concept, Zhang et al. [165] used sodium-rich nickel ferricyanide (NiHCF) and carbonyl organic compounds 5,7,12,14-Pentacenetetrone (PT) as the cathode and anode, respectively. They employed an ultra-high concentration of 17 M NaClO_4_ solution. Thanks to the low freezing point and high thermal stability of the “WISE” (Fig. 16a), an all-climate aqueous sodium-ion battery can be operated in a wide temperature range of − 40 to 100 °C. Electrochemical impedance spectroscopy (EIS) measurements indicated that the electrolyte maintains a relatively high ionic conductivity of 4.4 mS cm^−1^ even at − 40 °C (Fig. 16b). When decreasing to − 40 °C, the full cell can still display a discharge capacity of 85.6 mAh g_PT_^−1^ at a current density of 0.5 A g_PT_^−1^, remaining 68.7% of the room temperature capacity (Fig. 16c). Moreover, the constant discharge/charge profile of the battery within the temperature range of 25 to 100 °C revealed a slight increase in capacity with rising operating temperature (Fig. 16d). However, the high-temperature performance of the battery has not been further tested in this paper, and the mechanism of the operation of water in the electrolyte at high temperatures has also not been deeply explored, leaving room for further research in these areas.

Similarly, the strategy of using a high-concentration salt electrolyte is also applied in aqueous potassium-ion batteries. Jiang et al. [142] designed an aqueous potassium electricity operating in a wide temperature range of − 20 to 60 °C. By reducing free water molecules and water activity in the electrolyte, a wide ESW of 3 V was achieved (Fig. 16e). However, the ionic conductivity of the ultra-high-concentration electrolyte significantly decreases at − 30 °C, but remains relatively high at about 10 mS cm^−1^ and maintains lower viscosity at − 20 °C (Fig. 16f). When the temperature of the assembled pouch battery drops from 25 to − 10 °C, the capacity at a low discharge rate of 0.5 C decreases from 11.1 to 9.2 mAh. Subsequently, as the temperature rises to 60 °C, the capacity rebounds to 10.7 mAh. Despite the rate performance being unstable at 60 °C under 0.5 C, the battery exhibits very stability at 0.1 C under − 10 and − 20 °C with the coulomb efficiency close to 99.9% (Fig. 16g, h). Additionally, various inorganic salts with different anions, such as Cl^−^ [43] and BF_4_^−^ [166], are being explored as low-temperature additives; this approach provides valuable insights for the future development of AAMIBs.

Highly concentrated salts effectively reduce the activity of free water and enhance the solution’s water retention capacity by increasing the proportion of strong H-bonds. Additionally, as salt concentration increases, more free solvent molecules coordinate with metal ions, requiring greater energy to disrupt the solvation structure during evaporation. This process inhibits electrolyte volatility and enhances thermal stability [167, 168]. However, the high-concentration salt strategy remains constrained by cost and viscosity in practical applications. The ultra-high salt concentration results in electrolytes accounting for an excessive proportion of the overall cost, severely hindering scalability. Furthermore, viscosity plummets under extreme temperatures, leading to a severe deterioration in ion transport kinetics. This consequently compromises the stability of both the electrolyte system and the interfacial layer. Future research directions need to explore beyond the singular concentrated-salt approach, investigating moderate-to-low concentration mixed salts coupled with multifunctional additives to strike a balance between cost and stability.

### Organic Additives Strategy

**Fig. 17:**
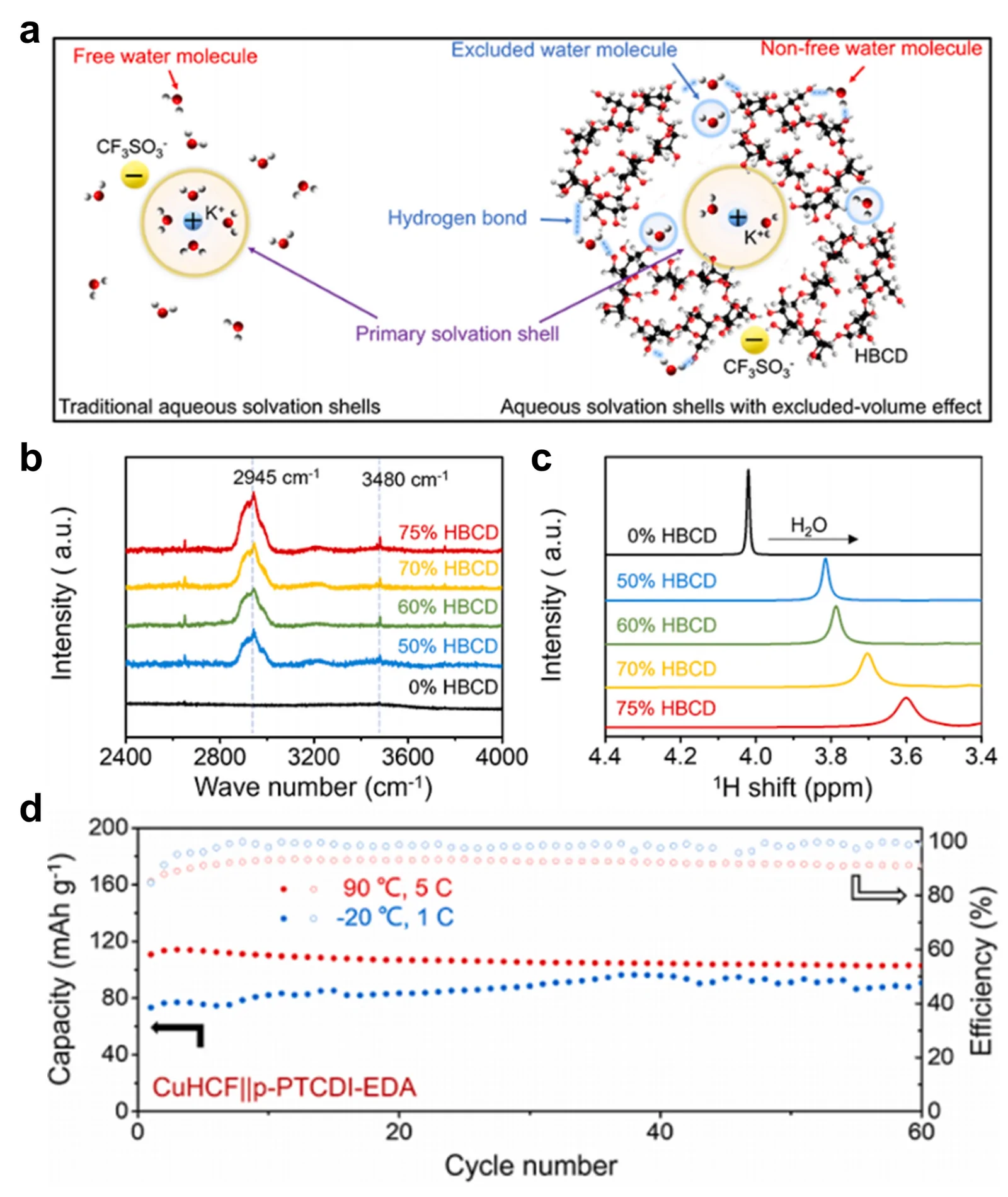
**a** Schematic illustration of K^+^ solvation shells in traditional aqueous electrolytes (left) and HBCD excluded-volume electrolytes (right), respectively. 2 M KCF_3_SO_3_-x HBCD-(1 − x) H_2_O electrolytes with different contents of HBCD (x = 0%, 50%, 60%, 70%, and 75%): **b** Raman spectra, **c** ^1^H NMR spectra. **d** Cycling tests of the CuHCF//p-PTCDI-EDA aqueous potassium-ion batteries at low temperature (− 20 °C, 1 C) and high temperature (90 °C, 5 C), respectively. Adapted with permission from Ref. [143]. Copyright 2023, Elsevier

The “volume exclusion effect” refers to a phenomenon in which larger particles occupy more space in the mixture, thereby limiting the distribution and interaction of smaller particles. This effect can reduce the homogeneity of the mixture. By using the synergistic effect of this effect and the hydrogen bond network, the ESW of the mixed electrolyte can be expanded and the activity of water molecules can be greatly reduced. This approach is crucial for improving the performance of these batteries across a wide temperature range. Wei et al. [143] introduced (2-hydroxypropyl)-β-cyclodextrin (HBCD), highly water-soluble, electrochemically inert, and low-cost supramolecular materials, into a 2 M KCF_3_SO_3_ aqueous electrolyte. HBCD is amphiphilic, featuring hydrophobic cavities and hydrophilic surfaces, and its volume is approximately two orders of magnitude than that of water molecules. This large size induces a significant “volume exclusion effect”. Additionally, HBCD has abundant hydroxyl side groups that facilitate the formation of a rich and dense hydrogen bond network with surrounding water molecules (Fig. 17a). In the traditional aqueous potassium-ion battery electrolyte, free water will preferentially form a solvation structure with K^+^, leading to side reaction of hydrogen evolution and oxygen evolution, which limit the operating voltage. The introduction of HBCD induces the “volume exclusion effect”, where free water near the solvated structure is repelled and confined within the limited space of HBCD. This significantly reduces the free water content in the electrolyte. Furthermore, the hydroxyl group of HBCD forms a dense hydrogen bond network with water, reducing water activity under the synergistic effect of these two factors. When the content of HBCD is increased to 75%, the ESW can reach more than 3.4 V. Raman spectrum (Fig. 17b) shows a reduction in the peak corresponding to O–H bonds at 3480 cm^−1^, indicating that the introduction of HBCD can significantly reduce the activity of water molecules and disrupts the water network connected by H-bonds. Additionally, with increasing HBCD content, the ^1^H NMR shift of H_2_O decreases (Fig. 17c), indicating that the electron density around the H atom of the H_2_O molecule is higher. This shift is likely due to the H-bond interaction between the O atom of HBCD and the H atom of H_2_O. As shown in Fig. 17d, at a low temperature of − 20 °C, aqueous potassium-ion batteries using this electrolyte still maintain a specific capacity of 80 mAh g^−1^ at 1 C, with nearly 100% coulombic efficiency, demonstrating that HBCD effectively supports low temperatures operation by inducing the volume exclusion effect. At a high temperature of 90 °C, the CuHCF//p-PTCDI-EDA aqueous potassium-ion batteries maintain a specific capacity of 120 mAh g^−1^ at 5 C, even higher than that tested at room temperature, indicating that higher operating temperature can improve the conductivity of K^+^ and active material utilization. This study provides a new approach to developing AAMIBs with wide ESW, high stability, and temperature resistance.

Organic additives enhance the performance of aqueous batteries at elevated temperatures through various mechanisms, including improving thermal stability, facilitating the formation of a stable SEI layer, and preventing electrode material dissolution [169]. However, organic additives are typically non-biodegradable and toxic, posing environmental risks that contradict the eco-friendly premise of aqueous batteries. Additionally, these additives may dilute local salt concentration, weakening the protective effect of the anion-derived interphase layer. Furthermore, certain additives may degrade or volatilize during temperature variations, causing their beneficial impacts to diminish over time. This consequently leads to electrolyte component imbalance and long-term performance degradation. Future research could focus on developing low-toxicity, bio-based, and eco-friendly alternatives (e.g., ionic liquid derivatives). In the recent research, Xu et al. [170] designed an aqueous electrolyte based on low-toxicity guanidine sulfate (Gdm_2_SO_4_) induced ion-water aggregates. This aggregate fixes anion migration and decouples metal cations from the solvation sheath simultaneously, achieving a high cation migration number, high ionic conductivity, and low solvation energy. It provides a new idea for low-cost, wide-temperature-range AAMIBs. Alternatively, designing dual-functional additives (e.g., fluoroether-based additives) that simultaneously improve freezing points and enable film formation could facilitate in situ construction of dense electrode interphase layers. Therefore, a comprehensive approach is required to develop optimized strategies for incorporating organic additives.

### Hydrogel Strategy

**Fig. 18:**
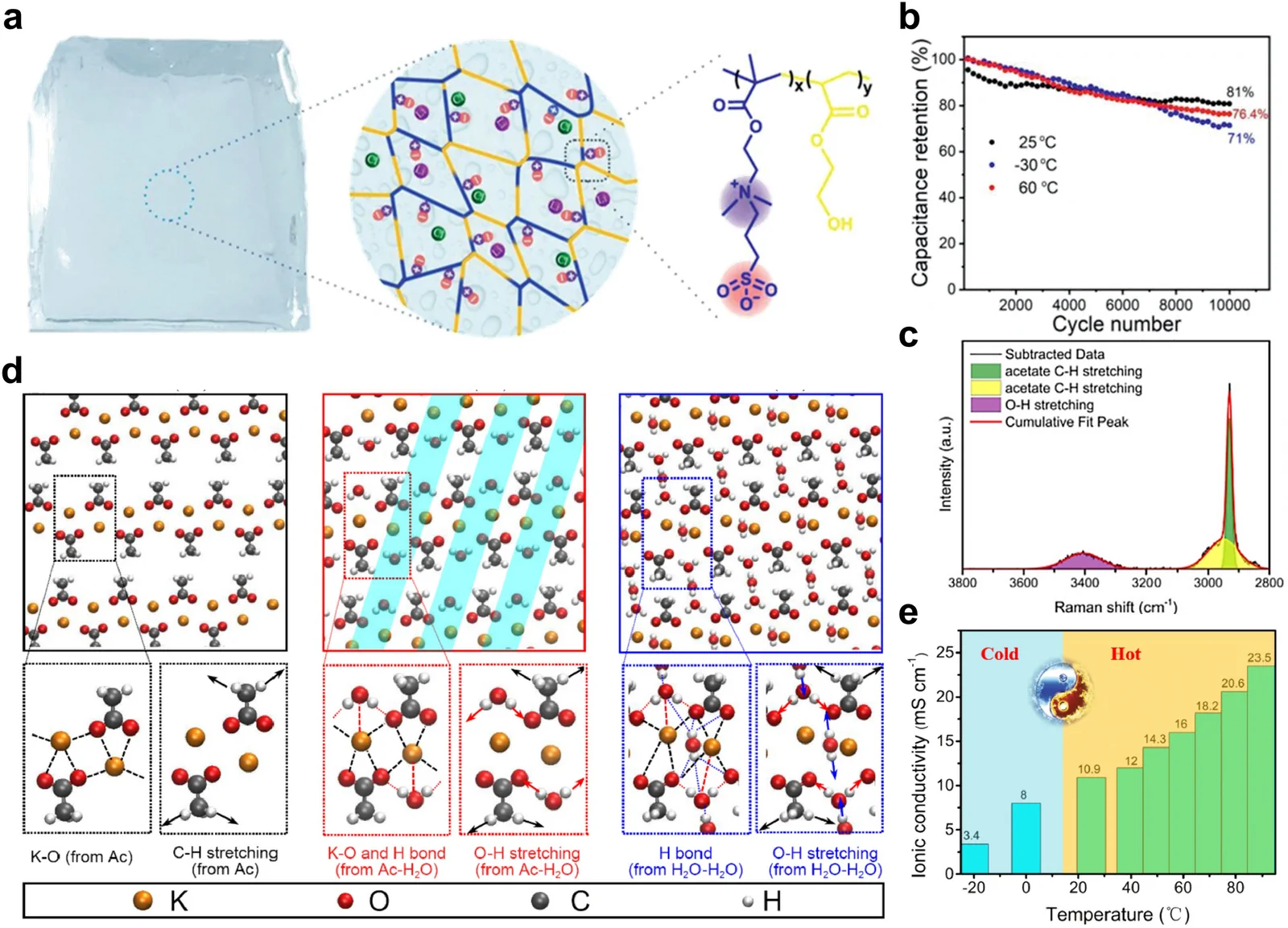
**a** Schematic diagram of anti-freezing polySH hydrogel and its network structure. **b** Cycle stability of the polySH-based SCs at different temperatures after 10,000 cycles. Adapted with permission from Ref. [135]. Copyright 2021, Wiley–VCH. **c** Raman spectra of the electrolytes with 48 m KAc gel in the O–H stretching and acetate C-H stretching vibration range. **d** A slice of DFT optimized structures of (left) pure anhydrous KAc powder, (middle) KAc/H_2_O = 1 (~ 48 m KAc gel), and (right) KAc/H_2_O = 1/2 (~ 30 m KAc gel); details of structures are expanded below; ionic and hydrogen bond are represented in dashed lines and vibrations are displayed in arrows. K, O, C, and H atoms are represented in orange, red, gray, and white balls, respectively. **e** Ionic conductivities of the 48 m gel at different temperatures. Adapted with permission from. Adapted with permission from Ref. [171]. Copyright 2021, Elsevier

Hydrogel electrolytes have been widely used and rapidly developed due to various advantages such as their flexibility, ease of fabrication, liquid-like ionic conductivity, and wide ESW. To improve the performance of hydrogel electrolytes at extreme temperatures, a variety of methods have been used, such as ionic liquids, polymers, highly concentrated electrolyte solutions, and organic solvents [151]. By simulating biological macromolecules, zwitterions, a new type of materials that contain cations and anions on the same unit, can be used as ideal materials for constructing anti-freezing hydrogels. Motivated by the above findings, a new type of antifreeze polymer (polySH) hydrogel electrolytes was designed and fabricated by random copolymerization of zwitterionic monomer (SBMA) and 2-hydroxyethyl acrylate (HEA) in the presence of LiCl salt (Fig. 18a) [135]. Owing to the synergistic effects including the electrostatic interactions between zwitterionic groups and Li^+^ as well as the formation of Li^+^(H_2_O)_n_ hydration structure, the polySH electrolyte exhibits an ultrahigh ionic conductivity of 12.6 mS cm^−1^ at − 40 °C. Due to its excellent water retention, hydrogel electrolytes also have good stability at high temperatures. The polySH-based SC exhibits a high specific capacitance of 178 mF cm^−2^ at 60 °C and 134 mF cm^−2^ even at − 30 °C with a retention of 81% and 71% of the initial capacitance after 10,000 cycles, respectively (Fig. 18b). In general, polymeric gelling agents are difficult to dissolve homogeneously in highly concentrated brines to prepare the gel state, and even when gels are obtained by fusion with a reduced salt ratio, the ionic conductivity of such electrolytes is usually not high. Based on this, Liu et al. [171] designed and prepared a KAc gel electrolyte without polymer additives, which takes into account the ionic conductivity while realizing the low activity of water molecules. In addition, the formation of the SEI layer further guarantees the stability of the active material during the repeated storage/release of potassium ions. The structure and bonding form of the resulting KAc gel system was explored by Raman fitting (Fig. 18c) and DFT theoretical calculations (Fig. 18d). It was found that the water in the 48 m KAc gel electrolyte mainly exists in the form of the NHW, basically there are no water molecules aggregated into water molecule clusters, and all the water molecules are bonded with either Ac^−^ or K^+^ to form hydrogen and ionic bonds, respectively, and the same interactions exist between Ac^−^ and K^+^, and the three of them, H_2_O, Ac^−^, and K^+^, are constructed into long polymer-like chains under the action of these chemical bonds, thus forming the cross-linking structure is similar to that of traditional hydrogels. Meanwhile, the ionic conductivity of the electrolyte can still reach 3.4 mS cm^−1^ at a low temperature of − 20 °C, and 23.5 mS cm^−1^ at a high temperature of 90 °C (Fig. 18e), and still maintains the quasi-solid state. Moreover, the high salt-to-water ratio enables the electrolyte to reach an ESW of 4 V, demonstrating excellent electrochemical stability and temperature adaptability. Hydrogel materials enhance the thermal stability of batteries due to their superior water retention capacity and contribute to structural integrity maintenance, owing to their excellent tensile and mechanical strength [172]. However, the polymer framework impedes effective electrode/electrolyte contact, resulting in charge transfer impedance significantly higher than that of organic systems. Future innovations must focus on interface engineering and growing conductive polymers on electrode surfaces to establish dual continuous ion/electron transport channels, thereby enhancing effective transport at the electrode/electrolyte interface. Additionally, the preparation of hydrogel electrolytes is typically complex, requiring precise control over polymer network formation and crosslinking density [36,171]. Future efforts should seek simpler processing methods.

## Conclusions and Outlook

**Fig. 19:**
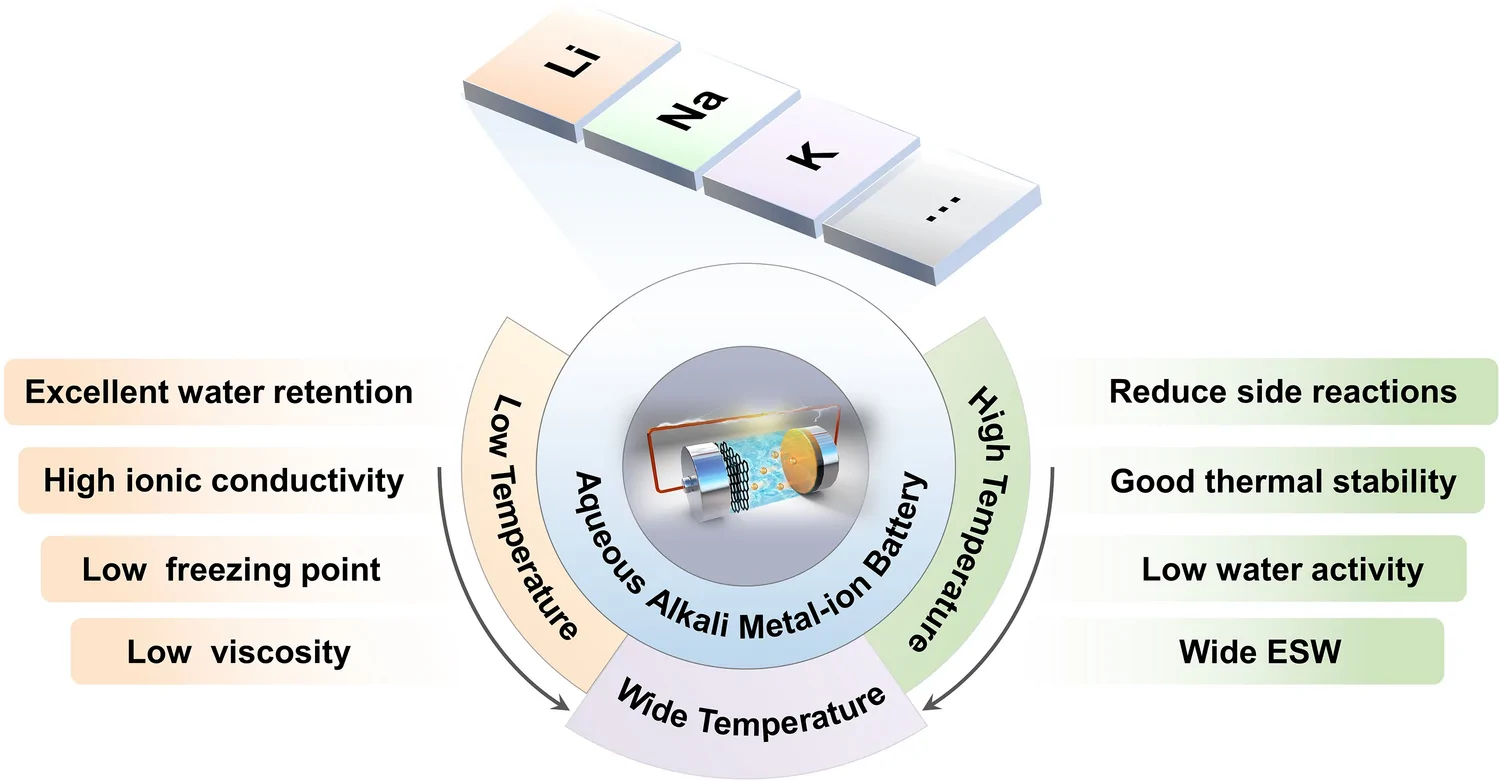
Design requirements for wide-temperature aqueous alkali metal ion batteries

In summary, this review provides a comprehensive analysis of recent research advances in wide-temperature electrolytes for aqueous alkali metal-ion batteries. We systematically examine the challenges faced by aqueous electrolytes under extreme temperature conditions and present a detailed discussion of the fundamental principles governing wide-temperature aqueous electrolytes, with particular focus on H-bond interactions in water molecules. Furthermore, we critically analyze various modification strategies. Compared with that of organic-based wide-temperature batteries, although the research on wide-temperature AAMIBs is still in its infancy, there have been some research cases that have successfully broadened their temperature range. The approach to improving the low-temperature aqueous electrolyte involves introducing substances that interact strongly with water molecules. This interaction disrupts the original hydrogen bond network, thereby lowering the freezing point of the electrolyte, increasing the ionic conductivity at low temperatures, and improving the charge transfer efficiency of the electrode/electrolyte interface. For high-temperature electrolytes, it is essential to manage the electrochemical activity and water retention to maintain stability and functionality at elevated temperatures. Based on these strategies, future research on high-performance wide-temperature aqueous alkali metal ion batteries can be further considered from the following aspects (Fig. 19).

1. **“WISE” Concentration Modulation** While high concentration electrolytes can broaden the ESW, lower the freezing point, and achieve higher energy density battery systems. However, a high concentration of electrolyte will not only increase the viscosity of the solution, but also greatly increase the cost, and cannot reflect the advantages of low-cost aqueous batteries. For this reason, the following ideas are proposed: (I) Seeking cheaper and more soluble salt; (II) Ensuring wide ESW and wide temperature range at low concentration by mixing multiple electrolytes. (III) Currently most H-bonds in H_2_O are interrupted by anions forming strong bonds with H, and it is considered whether there are suitable ions that can combine with OH to break H-bonds.
2. **Balancing Safety, Environmental Protection and Performance** The use of organic co-solvents and hydrogels can broaden the temperature range of aqueous electrolytes. However, the flammability of organic solvents could compromise the inherent safety of AAMIBs. Therefore, it is essential to carefully balance the ratios of salts, water, and organic solvents to maintain safety while optimizing electrochemical performance across different temperatures, particularly considering safety issues due to internal heating at high temperatures. In addition, most of the current high-concentration salt electrolytes use organic lithium salts containing elemental fluorine as the electrolyte, and the strong interaction between the F atom and water molecules realizes a low freezing point with a wide ESW. However, fluorine-containing electrolytes are expensive and pollute the environment, so inorganic salts with lower cost and greener environment should be used in the future to design high-concentration salt electrolytes, which destroys the intermolecular H-bonds of water through the interaction of anion and cation with water molecules and inhibits the activity of water.
3. **Interface Properties and Performance** From the kinetic aspect, increasing the HER and OER overpotentials by promoting the generation of the SEI/CEI layer on the positive and negative surfaces can serve to reduce the probability of water electrolysis. Current research primarily focuses on how interface-related impedance affects battery performance at low temperatures. Future work should delve deeper into interface properties, combining electrochemical experiments, theoretical calculations, and characterization tests to explore the solvation structure and desolvation processes.
4. **Integrated High-Temperature and Low-Temperature Performance** Research should aim to develop AAMIBs that perform well across high and low temperatures rather than address these temperature extremes in isolation. Compared with the research on low-temperature performance, the exploration of high-temperature-resistant AAMIBs is significantly under-explored. For applications at high temperatures, firstly, it is necessary to overcome the safety issues caused by internal heating of the battery at high temperatures, and secondly, it is necessary to inhibit the water activity to avoid violent side reactions at the interface. The selection of suitable inorganic salt solvents or organic co-solvents with intrinsic flame retardant properties can not only expand the ESW and inhibit the side reactions at the electrode surface by decreasing the water activity but also eliminate the safety hazards at high temperatures.
5. **Multi-Strategy Collaborative Optimization** The modification effect of a single strategy on the electrolyte is limited. The future breakthrough direction should focus on the deep integration of multi-strategy collaborative optimization and dynamic interface control. For example, introducing economical and environmentally friendly organic co-solvents or functional salt additives into medium and low-concentration salt electrolytes can achieve a multi-element synergistic system and regulate the solvation structure from multiple perspectives. In addition, materials containing specific elements can also be sought based on the system, and attempts can be made to design an adaptive repair interface layer to spontaneously form a healing network at the interface defects.
6. **Electrode Modification** In addition to electrolyte modulation, modifying electrode materials is crucial for enhancing the temperature tolerance of aqueous batteries. The lack of electrode materials with suitable potentials and the ease with which these materials dissolve in aqueous electrolytes pose a challenge. In terms of Prussian blue cathode materials, to reduce costs, protect the environment, and respond to the call for sustainable development, it is necessary to synthesize them with a gradient to achieve a substitution ratio in less Mn or Co while ensuring good electrochemical performance. As for anode materials, new organic materials with higher capacity and lower potential need to be further developed.
7. **Inactive Materials Modification** Although inactive materials such as diaphragms, collectors, conductive agents, and binders, they are not directly involved in the chemical reaction, and they affect the electrochemical performance of the battery by influencing the charge transfer resistance. The selection of diaphragms with high permeability, collectors with good contact, and conductive additives with excellent electrical conductivity all contribute to the rapid transport of ions at the interface.

Finally, while most current research focuses on aqueous lithium-ion batteries, the abundant reserves of sodium and potassium make aqueous sodium-ion and aqueous potassium-ion batteries attractive for future development due to their lower costs and potential wide temperature range. Moreover, hydrated sodium and potassium ions have smaller ionic radii and thus faster ionic transport rates. The future development of wide-temperature-range aqueous sodium-ion batteries and aqueous potassium-ion batteries with lower cost is also of great significance.
